# Status quo bias drives public support for drug policy in the USA

**DOI:** 10.1038/s41562-026-02471-y

**Published:** 2026-05-20

**Authors:** Bence Hamrak, Gabor Simonovits

**Affiliations:** 1 Department of Government, University of Vienna, Vienna, Austria; 2 Department of Political Science, Central European University, Vienna, Austria; 3 ELTE Centre for Social Sciences, Institute for Political Science, Budapest, Hungary

**Keywords:** Human behaviour, Politics and international relations

## Abstract

Substance regulation in the USA is in a crisis. Here we explore whether seemingly misaligned policies contradict public preferences or reflect biased citizen opinions. Using survey data (*N* = 5,053), we show that policy outcomes align with public opinion. We further demonstrate that regulatory preferences are driven by status quo bias: support for the legality of legal substances exceeds that for hypothetical drugs with corresponding properties (Δ_median drug_ = 39.33, 95% confidence interval 35.34 to 43.20), while the opposite holds for illegal drugs (Δ_median drug_ = −12.59, 95% confidence interval −15.44 to −9.87). Randomly assigning individuals to information about harm attenuates the status quo bias in the case of legal substances (Δ_median drug_ = −17.70, 95% confidence interval −22.35 to −13.05) but not for illegal ones. We discuss implications for public control over drug policies.

## Abstract (web summary)

Support for drug policies reflects status quo bias: the public favours legal drugs more than hypothetical drugs with identical properties. The reverse holds for illegal drugs. Harm information reduces this bias for legal drugs, but not illegal ones.

## Main

Current American drug policies are crumbling and leading to social outcomes that are suggesting regulatory failure. The policies in place to solve the abuse of psychoactive substances—commonly referred to as the ‘War on Drugs’—have led to mass incarcerations of predominantly Black men^1^, while failing to prevent the deaths of over a million people in the currently unfolding opioid epidemic^2^. Perhaps, more fundamentally, there appears to be a profound mismatch between expert opinion on the harms attributed to various substances and the spectrum of regulatory instruments applied to them^3–5^, driven by a history of cultural, racial and institutional bias^6–8^.

It is notable that while substance regulation might be seen as a public health issue that is exclusively in the hands of policy-makers and experts, the current status quo is not insulated from the public. There have been more than 25 state-wide ballots in the USA regarding substance regulation in the past 10 years, and overall, more than 100 million Americans had the chance to vote on these questions. Moreover, some studies indicate a growing liberalism of the drug policy mood of Americans^9^. Thus, any explanation of how such ineffective and unjust policies could have survived democratic control needs to account for the role of the public. Existing studies have shown that even though policy outcomes in general tend to respond to public opinion^10–12^, there is also evidence that owing to the perceptual and ideological biases of policy-makers or interest group pressure^13–15^, in some cases they remain incongruent with majority preferences. Finally, some studies also point to how various cognitive and affective biases lead citizens to support bad polices and how pandering to these preferences can lead to ineffective outcomes^16,17^.

The goal of the present Article is to explore the complex interplay between drug policies and public attitudes towards regulations. We argue that the legal status of a substance can shape public opinion that cements support for existing policies through a set of distinct mechanisms, which we call status quo bias. For example, the criminalization of a substance will lead to an over-representation of marginalized groups among its users. In turn, that will lead to the majority of the public only learning about the substance through media portrayals predominantly focusing on abuse and drug-related crime—rather than first-hand experience with either the substance or its users. Paradoxically, this could lead to both misperceptions regarding the harms associated with substance use and a misattribution of the malaise of drug use to the users or substances rather than to the criminalizing policies themselves^18^. Our conceptualization of the status quo bias is different from other accounts in psychology, which refer to a cognitive bias that leads individuals to be more risk avoidant in their choices if a baseline reference value is available^19,20^ or when the alternatives are framed as gains^21^.

We build on theories that highlight the link pointing from policy changes to public opinion^11,22–24^ and the susceptibility of citizen preferences to elite cues^25^. In this framework, our argument places a central emphasis on the lack of information citizens have about how policy instruments work. This argument corresponds to earlier work on the reliance of citizens to political cues^26–28^ and to research on the expressive, risk- or norm-signalling function of laws^29,30^. The pattern that public support can be maintained for policies that are ineffective simply because citizens rely on heuristics^26,27,31^ is visible in how voters reward incumbents for more visible disaster relief spending but not for preparedness efforts^16^, or how citizens, who have no direct experience with criminal justice, are more likely to rely on media portrayals to obtain knowledge^32^.

Our account also provides some hints regarding possible ways in which the public’s preferences could be enlightened. If citizens were to prioritize harms when evaluating drug polices, then their reliance on status quo cues could be countered by accurate information. There is a rich literature showing that citizens update their beliefs, political and policy preferences and attitudes upon receiving nudges for accurate thinking, arguments or factual information, even despite their initial beliefs^33–36^, even in the case of polarizing issues such as gun control^37^. However, it also remains possible that the misalignment of policies with substance harms in fact reflects affect and prejudice towards social groups^38^ who are over-represented among the users of a substance.

Unfortunately, existing studies are limited in their capacity and scope to inform us about the factors shaping public opinion on substance regulation. Past research focuses on special populations or measures public opinion on individual drugs with idiosyncratic features^39–43^. Yet others combine multiple polls to estimate changes in the public’s ‘general mood’ about drug policy^9^. While this literature has provided important insights about public opinion, it does not allow a comparison of a wide range of substances with varied harms and legal status and is not amenable to address either the link between substance harm and support for legalization, or the role of the status quo in shaping public opinion.

Our empirical analysis, in turn, relies on a methodology that permits us to address these challenges. First, we recruited a sample of *N* = 2,016 participants from Lucid, an online opt-in panel that has shown to produce results that resemble probability samples in terms of demographics^44^. In the survey, we elicited support for the legality of 13 common, currently legal and illegal substances that vary in terms of their harm. Then, we matched the resulting estimates of policy support with expert classification of substance harm^5^ (Methods and Supplementary Table 1). The resulting data permit comparisons between public preferences for regulation across different types of substances as well as a measure of correspondence between drug harm and public support for legality.

To explore preference formation in the absence of the status quo bias, we rely on an experiment embedded in the same survey that measures support for the legality of hypothetical substances characterized by their randomly assigned harm potential as well as other attributes (Methods). This approach allows us to explore the impact of the status quo through the comparison of preferences for real substances, and their off-brand versions, defined as hypothetical substances with a combination of attributes (for example, physical harms to the user) that reflect the properties of each real drug we study. Moreover, the experimental data also allow us to assess what attributes make the public opposed to legalizing certain substances but not others by estimating the impact of drug harms vis-a-vis non-harm-related considerations, such as the purpose of substance use. Finally, we conducted a follow-up study (*N* = 3,037) recruited through the same survey vendor in which we both measured and corrected citizen perceptions to explore the potential of providing better-quality information on harms on the preferred legality of various substances.

## Results

**Fig. 1:**
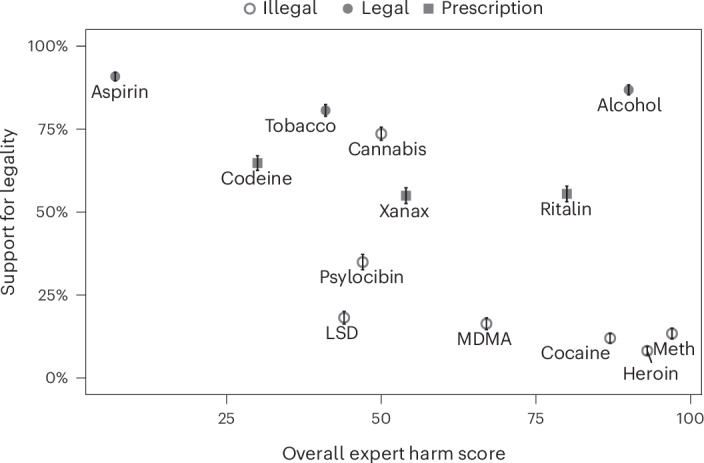
Expert scores for drug harm and respondent support for legality (binary). The harm scores range from 0 to 100. For details on harm expert scoring, see the Methods and Supplementary Table 1. In case of support, respondents rated only familiar substances, therefore *n* varies by substance (*n*_Alcohol _= 1,930; *n*_Aspirin _= 1,893; *n*_Cannabis _= 1,917; *n*_Cocaine _= 1,817; *n*_Codeine _= 1,816; *n*_Heroin _= 1,780; *n*_LSD _= 1,595; *n*_MDMA _= 1,662; *n*_Meth _= 1,702; *n*_Psylocibin _= 1,643; *n*_Ritalin _= 1,727; *n*_Tobacco _= 1,931; and *n*_Xanax _= 1,700). Support is summarized with mean percentages and 95% CIs.

We motivate our exploration of status quo bias by first comparing mean support for the legality of recreational use across several currently legal and illicit substances. Figure 1 plots respondent support for legality against expert-rated overall harm. The association between harm and support is negative but not statistically significant (*r* = −0.54, 95% confidence interval (CI) −0.84 to 0.02, d.f. of 11, *P* = 0.060). This lack of statistical significance is partly driven by notable deviations from the overall pattern. In particular, respondents express substantially higher support for the legality of alcohol than for 3,4-methylenedioxymethamphetamine (MDMA) (Δ = 71.08, 95% CI 68.61 to 73.54, d.f. of 1,638; paired *t*-test, *P* < 0.001), even though MDMA is rated by experts as less harmful overall. Such cases challenge a simple harm-based account of legalization preferences. Instead, the clustering of substances by their current legal status is consistent with the possibility that existing regulations shape public preferences, rather than harm assessments alone determining support.

### Quantifying the bias in public opinion for drug support

**Fig. 2:**
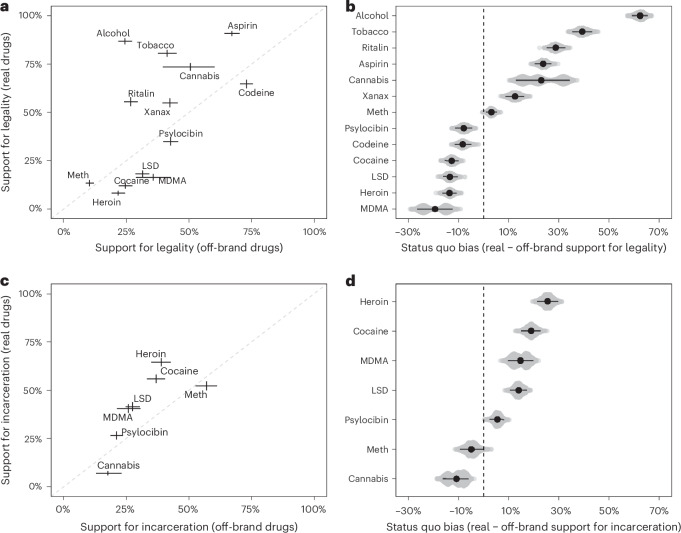
Respondent support for legality (binary) for real and off-brand substances. **a**, The bootstrapped (1,000 resamples; original *n* = 2,016 respondents) mean support (percentages) with 95% CIs for legality. **b**, The mean support gap between real and off-brand substances across bootstrap samples for legality. **c**, As in **a**, but showing support for incarceration for illicit substance use (binary). **d**, As in **b**, but showing support for incarceration for illicit substance use (binary). Overlaid raw data indicate the full bootstrap sampling distribution. For substances with mixed neurological effects, multiple mean estimates were averaged. Numerical details are shown in Supplementary Tables 4 and 5.

To quantify the impact of the status quo, we first estimate the legalization preferences for the off-brand version of each drug. We define this as the experimentally predicted support for a hypothetical drug profile that matched the attributes of the same real substance. In Fig. 2a,b we show the differential support for each of the 13 real drugs with their off-brand versions. In the case of substances that are currently available for recreational use on the federal level, support is much larger as compared with their off-brand counterparts. Bootstrapped means for the differences are consistently positive for three legal substances (aspirin, tobacco and alcohol), ranging from 23.8 to 62.4 percentage points (95% CI 20.5 to 65.4).

Respondents also exhibit a positive bias for prescription drugs such as amphetamines and Xanax (between 12.5 and 28.8 percentage points, 95% CI 8.8 to 32.5)—with the exception of codeine ($\overline{x}$= −8.16, 95% CI −11.3 to −4.7). Similarly, we find a positive bias for cannabis ($\overline{x}$= 23.0, 95% CI 12.9 to 34.2)—a substance that is illegal on the federal level but available for recreational use in most US states. By contrast, there is a corresponding, albeit smaller bias, pointing to the opposite sign in the case of almost all illegal drugs, even in the case of psychedelics, which have comparatively little harm potential. Five illicit substances are less supported compared with their off-brand version (psylocibin, lysergic acid diethylamide (LSD), heroin, cocaine and MDMA), with their bias ranging from −19.4 to −7.8 percentage points (95% CI −26.5 to −4.5). The only exception is methamphetamine (meth), which respondents find even more harmful when evaluated off-brand ($\overline{x}$= 3.1, 95% CI 0.9 to 5.2). For the full list of substance-level results, see Fig. 2a,b and Supplementary Table 4.

To obtain a more complete picture of the possible scope of the status quo bias, in the case of currently illegal substances, we also assess the support for incarceration as a preferred punishment for substance use in Fig. 2c,d. This is measured as respondents’ preference for felony as a form of punishment for substance use in case of drugs they have preferred to be illegal. These tests were not preregistered, and we report them as exploratory analyses only. We find that survey respondents were generally less likely to support incarceration when the off-brand version of the drug was shown in the case of five illicit substances, suggesting that status quo bias may also explain support for punitive policies in the case of these drugs. This bias ranges between 5.5 and 25.5 percentage points (95% CI 2.4 to 29.7). Meth ($\overline{x}$= −4.8, 95% CI −9.3 to −0.3) and cannabis ($\overline{x}$= −10.7, 95% CI −16.2 to −5.9) are again exceptions—which is not surprising, given that they are also associated with a positive bias in support for legality. For the full list of substance-level results, see Fig. 2c,d and Supplementary Table 5.

A key finding from our experiment is that in the absence of status quo bias, most our sample would not support the legality of almost any of the substances (except painkillers), but the punitive policies that are currently in place for most illegal drugs are not supported by the majority either.

#### Effect of substance attributes on legalization support

The experimental set-up also allows us to discern how substance attributes determine support, uninfluenced by confounders such as the status quo. We use the data to quantify how much substance harm matters for support for legality vis-a-vis other, non-harm-based attributes in counterfactual set-ups where prior legislation does not exist. In other words, we can test how ‘objective’ public regulation preferences would be without the status quo bias. Since in the case of hypothetical drug profiles presented to the respondents these attributes were randomized and independent of each other, we can assess their causal impact on support. We did not formulate hypotheses about the effects of the attributes, and we report the results of an exploratory analysis.

**Fig. 3:**
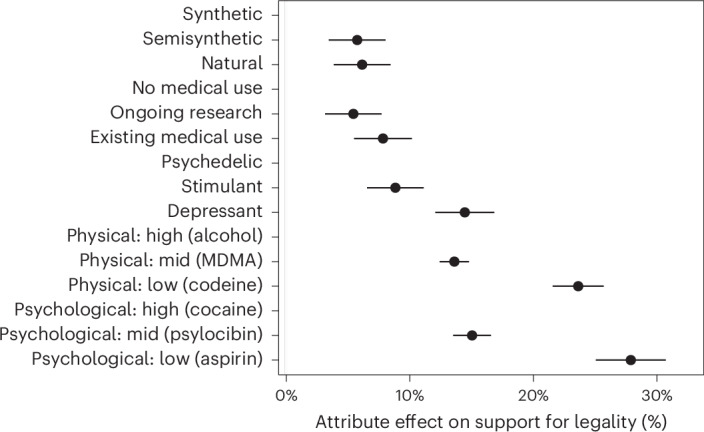
Drug attributes and hypothetical drug legalization support (*N* = 2,016). Point estimates are average marginal effects from a logit regression (Supplementary Information, section G) and show the percentage point change in mean respondent support for each orthogonally randomized levels of drug attributes compared with their reference category. For the harm attributes (0–100), coefficients are multiplied to show model-based predicted differences in support between low-, medium- and high-harm drugs (the reference category). The 95% CIs are clustered on the respondent level (multiple drug profiles per respondent). See the regression results in Supplementary Table 6.

Figure 3 shows that harms indeed play the most influential role in preference formation. Compared with alcohol—a substance representative of high physical harm—for example, a hypothetical substance with moderate physical harm such as MDMA or low physical harm such as codeine, is estimated to have on average 13.6 (*z*(9,981) = 22.41, 95% CI 12.4 to 14.8, *P* < 0.001, Cohen’s *d* = 0.28) and 23.6 percentage points higher legalization support (*z*(9,981) = 22.41, 95% CI 21.6 to 25.7, *P* < 0.001, Cohen’s *d* = 0.48), respectively, on the basis of estimates from our logit regression model (Supplementary Information, section G). However, holding harm profiles constant, people still report a strong distaste for psychedelic substances and favour depressants (*β* = 14.5, *z*(9,981) = 11.9, 95% CI 12.1 to 16.8, *P* < 0.001, Cohen’s *d* = 0.29), suggesting that social norms about the acceptable purposes of drug use also shape preferences.

While the main goal of this experiment is to characterize how factors that vary across drugs influence public support for their legality, we also explore individual-level heterogeneity in hypothetical drug support and the status quo bias. We report results exploring the main effect as well as moderating role of gender, race, educational attainment and partisanship in Supplementary Tables 7–11.

### The effect of harm information on the status quo bias

Given the evidence of status quo bias, our final objective is to assess whether providing accurate information can better align public preferences for substance regulation with expert opinion on harms. On the one hand, this is motivated by the results of the previous experiment, where we find that citizens indeed focus on harm when deciding about legality. On the other hand, the provision of information also presents an actionable policy alternative for governments or non-governmental organizations attempting to educate the public.

First, consistent with our arguments about the effect of the status quo cues, we find that citizens, in most cases, indeed underestimate substance harms for currently legal substances, and, in most cases, overestimate those for illegal drugs. For example, in the case of alcohol, respondents perceive physical and psychological harms on average −17.4 percentage points lower compared with the expert score (d.f. of 392, *P* < 0.001, Cohen’s *d* = −0.85). By contrast, for example, the subjective harm rating for MDMA is 20.6 percentage points higher compared with the expert score (d.f. of 411, *P* < 0.001, Cohen’s *d* = 0.95). See the detailed, substance-level results for the baseline harm misperception in Supplementary Table 12.

**Fig. 4:**
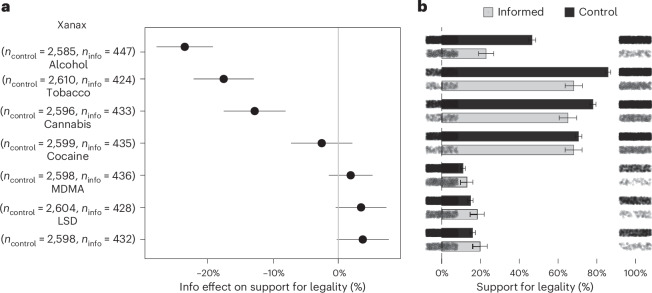
Harm information effect on drug legalization support. **a**, The mean differences in respondent support for a substance between the harm information treatment group and a control group who received no harm information (zero line). Estimates are based on two-sample, two-tailed *t*-tests. **b**, The absolute support levels per experimental groups with overlaid raw response data. Error bars denote 95% CIs. See numeric details in Supplementary Table 13. Xanax: *n*_control _= 2,585; *n*_info _= 447. Alcohol: *n*_control _= 2,610; *n*_info _= 424. Tobacco: *n*_control _= 2,596; *n*_info _= 433. Cannabis: *n*_control _= 2,599; *n*_info _= 435. Cocaine: *n*_control _= 2,598; *n*_info _= 436. MDMA: *n*_control _= 2,604; *n*_info _= 428. LSD: *n*_control _= 2,598; and *n*_info _= 432. Info, information.

However, in Fig. 4a,b, we show a clear separation of how accurate information about harms by experts shapes public opinion across legal versus illegal substances. Support for legalization is remarkably decreased for legal substances in the informed treatment condition compared with the no-information condition (control group), both in the case of alcohol (Δ = −17.7, 95% CI −22.3 to −13.1, d.f. of 502.53, *P* < 0.001, Cohen’s *d* = −0.48) and tobacco (Δ = −12.9, 95% CI −17.7 to −8.1, d.f. of 545.90, *P* < 0.001, Cohen’s *d* = −0.30). By contrast, citizens appear more reluctant to increase their support for illegal substances as a result of harm information. Change in legalization support is small and not statistically significant for any of the drugs. For a full list of substance-level comparisons, see Fig. 4a,b and Supplementary Table 13.

As the baseline harm misperceptions are similar across legal and illegal substances (when averaged over all drugs), this is unlikely to account for the asymmetry in citizens’ updating on legalization support owing to better information. We suspect that, instead, it is explained by the non-harm-related aspects of drugs and drug use. Our experimental evidence showing a strong preference against hypothetical substances with psychedelic effects is one such aspect. Other mechanisms such as the role of prejudices towards user groups^38^ remain unexplored.

Finally, we synthesize our findings on the impact of providing accurate information about harms on reducing the status quo bias (difference between real and off-brand preferences) in Supplementary Fig. 3. Overall, correcting misperceptions dissipates, on average, only 32% of the total status quo bias for legal substances characterized by a positive bias (alcohol and tobacco), and 18% for illegal substances characterized by a negative bias (cocaine, LSD and MDMA). Interestingly, Xanax, a prescription drug, is an exception: providing harm information results in support that is even lower than for the off-brand version. Speculatively, the combination of Xanax’s reputation as a medicine and information on its relatively high harms might create a backlash effect.

## Discussion

The key result that emerges from our research is that the public does prioritize harms when forming an opinion about the legal status of both actual and hypothetical psychoactive substances. However, it seems that status quo bias results in a mismatch between expert and lay opinion about drug policy. In the case of currently legal and harmful drugs, status quo bias manifests in a form of underestimation of harms: citizens update their views on legality once their harm perceptions are corrected. By contrast, in the case of relatively less harmful, but currently illegal drugs, preferences appear less sensitive to information about harm. The fact that factors unrelated to harm shape preferences too—at least in the case of hypothetical substances—suggests that the impact of the status quo also manifests in deeply held cultural norms about the acceptable forms of drug use that are divorced from drugs’ true harm potential^6,7,45^, and sustain punitive policies whose motives are entangled with systematic racism^46,47^.

An important limitation of our approach is our simplistic conceptualization of drug harms that implies that harms only depend on the properties of substances themselves but not on the characteristics of users, mode of use or the social context. Clearly, this misses some quite important facts about drug abuse: in reality, these latter factors do matter, and among other things, the legal status quo does contribute to the harms of drug use, for instance, by marginalizing the users of illegal substances. These limitations highlight the problem that expert data are too coarse to disentangle these sources of harm, because counterfactual harms under different legal regimes and environments are impossible to observe. Furthermore, knowledge about the causes of harm is not the only bottleneck for better policies: broadly applicable instruments, such as legalization or criminalization, are also too blunt to control factors such as dosage, making it difficult to design effective and just regulation.

Another limitation of our approach is our reliance on cross-sectional data on both public opinion, policy and expert ratings. Clearly, none of these variables are static, as probably best demonstrated by the history of tobacco that exemplifies abrupt changes in both expert opinion, regulation and the attitudes and behaviours of lay people. As a result, our findings are not informative of the possible dynamics of status quo bias, though it seems likely that the biases held by the public are the largest when regulation and true risks associated with substances are so out of step with each other. All in all, exploring how status quo bias might change over time, especially in the face of a seeming liberalization of public opinion towards some substances, appears to be an important avenue for future research.

Against the backdrop of our findings, a question remains whether the public should weigh in on policy decisions regarding substance regulation. Democratic theories highlight the importance of policy responsiveness, while empirical research often demonstrates that public pressure leads to suboptimal policy outcomes when leaders pander to the cognitive biases and prejudices held by the public^16,32^. In the meantime, it is unclear whether the insulation of drug policy from electoral pressure would lead to better outcomes given that policy-makers also exhibit various biases^13,14,48^, and in the absence of popular control, policy outcomes often fall prey to the pressure of organized interest groups. All in all, an equilibrium with drug policies more aligned with substance harms would require a combination of better information about harms and about the effects of regulatory instruments as well, and a less biased public discourse, inclusive to the perspective of users^49,50^. Ultimately, this would allow the public to better understand the relationship between regulations and their consequences so that they are in a better position to enhance accountability in this policy domain.

## Methods

### Substance data

First, we create a dataset of real substances and their attributes. We study a set of 13 common psychoactive substances that vary in terms of their legal status in the USA. We selected these drugs from a longer list of substances on the basis of the criteria that their familiarity exceeded 75% based on a pilot study (*N* = 1,767) (see more details in Supplementary Information, section B). The pilot study was exempt from the requirement for ethics review according to the policies of the Central European University.

Then, for each substance, we first extract a holistic evaluation of substance harm that includes both harms to the user and social consequences of abuse—for example, crime—that are potentially also influenced by the regulatory status quo. Moreover, we also separately consider physical harms to the user (organ impairment or harm related to injection or ingestion), and one related to the potential psychological harm to the user (mental impairment or addiction) caused by the consumption. The expert study that we rely on ranks these substances against each other (1–30, where the 30th drug was the least harmful) along these harm dimensions^5^. To calculate our own harm scores, we normalize and reverse the original rank score (1–30) to a 0–100 scale.

Beyond harm to the users, we also record the production source (that is, natural, synthetic or semisynthetic), and the therapeutic benefits associated with use (known medical benefits, no known benefits or under research). Finally, we also consider the substances’ effect on the brain, distinguishing between drugs with a stimulant, depressant or psychedelic effect on the nervous system. For some substances, we create mixed neurological effect profiles (for example, MDMA shares stimulant and psychedelic effects). Because even these seemingly objective data on substance attributes can be biased owing to pressure exerted by interest groups and the ideological bias of policy-makers^7^, we double-checked official information provided by authorities (such as the US Food and Drug Administration (FDA) or Drug Enforcement Agency) with alternative but verified psychiatric sources. For example, to categorize the therapeutic benefits of some psychedelic drugs, we overrule the FDA (https://www.fda.gov/drugs/development-approval-process-drugs/drug-approvals-and-databases) and Drug Enforcement Agency fact sheets (https://www.dea.gov/factsheets) using information from the Multidisciplinary Association for Psychedelic Studies and related research (https://maps.org/advancing-research/). We are fully transparent about the results of these decisions, and we share the details about the derived attributes of substances included in the study in Supplementary Table 1.

### Survey data

Second, we collected survey data to measure citizen preferences for the legal status of both real and hypothetical substances. The studies received ethical approval from the Ethics Committee of the Central European University (2022-2023/6/EX). Overall, we are confident that our research presented little to no harm to participants as we did not use deception and relied on hypothetical scenarios. Participants also read an informed consent form at the beginning of the surveys and were told that they are free to withdraw from the studies at any time (Supplementary Information, section D.1). Furthermore, participants were explained the consequences of their answers, and the goals of the studies in a debriefing (Supplementary Information, section E.5).

Our first survey was fielded through Qualtrics to an online opt-in panel matched to population census quotas (age, gender, race, ethnicity, and geographic regions) recruited through Lucid Theorem^51^ on 21 July 2023. Participants in the study were compensated according to their agreement with the sample provider. Altogether, 2,016 respondents completed our survey with an overall response rate of 82% (2,460 respondents attempted the survey). Attrition included failed consent form and failed attention check. No statistical methods were used to predetermine our sample size, but our sample size is larger than those reported in previous publications^42,52^.

Our survey was preregistered on 19 July 2023 (https://osf.io/7jn3t). We followed the exact analysis protocol regarding each preregistered hypothesis. We report the analysis of the size of the status quo bias for cannabis by state legislation type in the Supplementary Information of this article (Supplementary Fig. 4).

Respondents were first screened about their familiarity with each of the 13 substances. Then, they were asked whether they thought each of the substances they were familiar with should be legal (1) or illegal (0) for recreational use, and if respondents preferred illegality, we also asked their punitive preferences (decriminalization, misdemeanour or felony) (Supplementary Information, section D.2). Later, in an experimental block (Supplementary Information, section D.3), respondents were asked to imagine that they were drug experts who decide over the legal status of new hypothetical drugs—with information about attributes included in our benchmark data. They were first given the definition of these attributes (for example, that by physical harm we meant the severity of the possible organ damage resulting from activities related to consumption, such as ingestion) following the actual expert coding instructions for these harm scales^3–5^. Then, they were shown the profile of five hypothetical drugs and were asked to decide on the legality for recreational use, and, in case of illegality, also punitive preferences, as they did in the case of the real substances (Supplementary Information, section D.4). The attributes in the hypothetical substance profiles—physical and psychological harm, production source, neurological effects and medical use—were independently randomized (simple, double blind). We highlight a possible limitation in the design regarding the neurological effects of the experimental substances. To make the descriptions more vivid, we included information about some societal functions of types of substances alongside their pharmacological effects. For example, stimulants were described as ‘making users less tired’ and ‘enhance productivity’. Clearly, these are socially constructed purposes of substance use, which are also influenced by the status quo. However, if anything, these could have made the legalization support gap between the experimental and real drugs smaller. For example, the social function of psychedelics appears less accepted by citizens, while depressants—which are often legal substances—appear more accepted (Fig. 3).

The resulting data from the experimental tasks is first used to estimate the causal impact of individual attribute levels on the legalization support of hypothetical substances. We model these effects via a binomial logistic regression (Supplementary Information, section G) because of the binary outcome variable. Therefore, our estimates and predictions range from 0 to 1. For ease of interpretation, the log-odds are transformed to average marginal effects, so each coefficient can be interpreted as a percentage point change in support. We cluster standard errors on the respondent level because of the repeated measures (that is, individuals answered multiple profiles). With 80% power, we can detect treatment effects as small as 3.4 percentage points for the categorical attributes (source, medical use and effect), and 0.05 percentage points for a 1-unit increase in the continuous variables (harms; 0–100 range) at the 95% confidence level.

Then, using the same logit model, we predict support for the legality of off-brand versions of the studied substances. We define these as the experimental profiles that correspond to the attributes of a real-world substance, matched on the basis of the benchmark data. For substances with mixed neurological effects, we average predicted levels of support from each attribute profile variant.

Finally, we calculate the status quo bias for each substance as the simple difference between the real (from the pretreatment question) and off-brand ratings (based on the experimental predictions). For statistical inference, we rely on blocked bootstrapping. Specifically, we resample from the initial population of respondents, calculate both observed and modelled levels of support, and construct the 95% CIs on the basis of the corresponding values of the bootstrapped samples for each substance. We reject the null hypothesis (no status quo bias) for a specific substance if its CI crosses zero. We follow the same method to predict and compare the real and off-brand support for incarceration (0 if legal, or preferred punishment is not felony) of illegal substances.

### Follow-up survey data

We ran a follow-up experiment, aiming to test the role of harm information on the status quo bias. The survey was fielded on 23 April 2024 through Qualtrics and the same vendor, and using the same sampling and recruitment strategy as in case of the original survey. Overall, 3,037 respondents completed the survey, with an overall response rate of 83.5%. Attrition was due to respondents that failed to approve the consent form or failed attention checks (3,638 preconsent and attention check). No statistical methods were used to predetermine our sample size, but our sample size is similar to those reported in relevant publications^37,38^.

The experiment was preregistered on 28 March 2024 (https://osf.io/h6gwb). We follow the exact analysis protocol of the hypotheses, except for two minor deviations. First, we calculate the harm information treatment effect on legalization support using a two-sample, two-tailed *t*-test instead of bivariate ordinary least squares regression. In the case of simple treatment and control group experiments (without controls), the estimates and the inferences are identical between these tests. Second, we do not correct our estimates for multiple measurement when evaluating statistical significance because we test the hypotheses at the substance level, with independent subsamples.

We selected 7 out of the 13 substances of the original survey on the basis of their variations in the substance attributes. The survey had two outcomes: respondents’ support for recreational legalization (the same question as in the original study) and respondents’ perceptions of substance harms. For each respondent, we randomly selected one substance for a harm information treatment condition, and we showed them the actual expert rating of harms (based on the expert benchmark data) for that substance, using the same scale (0–100) and definitions as in the original study (Supplementary Information, section E.3). Then, we measured support post-treatment for this substance (the same as in the original study). For the rest of the substances (control condition), we measured support in a pretreatment question (the same as in the original study). In contrast to the previous study, participants also rated substances they were not familiar with on the basis of the screening question (the same as in the original study).

After creating substance-level subsets of the resulting data, first, we estimate the impact of harm information by comparing the mean support for legality among those respondents who were provided information about the given substance (treatment condition) and those who were not (control condition), using two-sample, two-tailed *t*-tests with 95% CIs. With 80% power, we can detect information treatment effects as small as 5.2 percentage points at the 95% confidence level.

Besides support for legality, we also measured respondents’ perception of harms (Supplementary Information, section E.2) for a randomly chosen substance that the respondent reported familiarity with but was not selected for the harm information treatment condition. To calculate the misperception of harm, we test the mean difference of the self-reported (subjective) harm perception and the actual expert harm scores, using one-sample *t*-tests. Moreover, we also measured the harm perceptions for the randomly selected treatment substance (informed condition) after the stimuli and support question. As a manipulation check, we compare the mean misperception (difference of reported harm and expert-assessed harm) under the treatment (informed) and control condition (uninformed) with two-sample, two-tailed *t*-tests with 95% CIs. All data analyses are performed with the statistical software R (version 4.4.2). For more details, see the packages used for the data analysis in the Reporting summary.

### Limitations

Here we discuss some limitations of our experimental design. A set of problems arise from the issue that the harms of drug use are determined jointly by both the properties of a substance, the characteristics of the user, the dosage and the mode of consumption. For instance, heroin, a drug that is typically injected, can also be ingested by smoking or injection, with the latter method carrying considerably higher risk. A more risk-seeking user may prefer injection as well as a higher dosage, which in turn carries substantial risks. Similarly, many of the psychological risks of psychedelics are moderated by pre-existing mental health issues of some users. These complications present three distinct issues for our research.

First, to the best of our knowledge, there exist no comprehensive expert data on the physical or psychological harms of substances as a function of these parameters. Thus, the available datasets—such as the one we rely on—present risk for an unspecified ‘prototypical consumer’, or possibly a worst-case scenario. This lack of granularity in our knowledge of drug harms presents a measurement problem: it is likely that cognitive biases and structural inequalities—such as the ones our paper discusses—become hardwired in the production of knowledge regarding substances. At the same time, this issue also points to the epistemological problem of generating knowledge regarding the impact of substances on humans in an environment where the legal framework as well as individual preferences lead to very clear and non-random selection of users to substances and substance use strategies.

A second issue that necessarily follows from the problem described above is that, in our own experiment, we needed to simplify our presentation of both real and hypothetical substances. This also resulted in the introduction of potential confounders in the experimental drug profiles (such as not specifying dosage levels). We accounted for this by operationalizing ‘harm potential’ rather than harms themselves. Therefore, we believe respondents had similar latent perceptions about a ‘worst-case scenario’, making their preferences comparable to each other. Of course, citizens’ typical dosage perception associated to real substances could be different. However, we argue this is also a sign of a status quo bias for legal or illegal substances (for example, over- or underestimating typical dosage).

It is clear that individual characteristics matter for the consequences of drug use, as does dosage and the mode of consumption. However, we note that the ultimate focus of our paper is drug policy, rather than drug consumption. Regulatory policies—with some limited exceptions, such as age limits on the sale of certain substances, or reliance of psychiatric diagnosis in the case of drugs available for therapeutic use—are necessarily blunt tools that cannot effectively enforce rules regarding dosage or the mode of consumption. Thus, we believe that while our approach surely introduces important simplifications, in some sense, it remains at the same level of granularity as public policies can be.

### Reporting summary

Further information on research design is available in the Nature Portfolio Reporting Summary linked to this article.

## Supplementary information

Supplementary Information Supplementary Tables 1–14, Supplementary Figs. 1–4, Supplementary Discussion and survey questionnaire.

Reporting Summary

## Data Availability

The data necessary to reproduce or replicate our analyses are available via Zenodo at 10.5281/zenodo.19130713 (ref. ^53^).

## Code availability

All computer code necessary to replicate our analyses is available via Zenodo at 10.5281/zenodo.19130713 (ref. ^53^).
