# Supplementary material for: Status quo bias drives public support for drug policy in the USA
Source: Nat Hum Behav. 2026 May 20;10(8):1487–94. doi: 10.1038/s41562-026-02471-y (PMC13485701; doi:10.1038/s41562-026-02471-y)
Supplement: Supplementary file 1 [file 41562_2026_2471_MOESM1_ESM.pdf]

nature human behaviour

Supplementary information https://doi.org/10.1038/s41562-026-02471-y

Status quo bias drives public support for drug policy in the USA

In the format provided by the authors and unedited

Contents

A Substance attributes and harm data **3**

B Pilot study **4**

C Sample characteristics **5**

D Survey wording **6**

D.1 Informed consent form and attention check 6

D.2 Pre-treatment items 7

D.3 Experimental stimuli 8

D.4 Post-treatment items 10

E Follow-up survey wording **11**

E.1 Pre-treatment items 11

E.2 Subjective harm rating task 12

E.3 Experimental stimuli 12

E.4 Post-treatment items 13

E.5 Debrief 14

F Pre-registration **15**

G Attribute effects and off-brand substance support prediction model **15**

H Status quo bias tests **16**

I Experimental substance attribute effects **17**

J Respondent heterogeneity in experimental substabce attribute effects and the status quo bias **17**

K Harm information effects **24**

L Status quo bias moderation by harm information **25**

M Status quo bias for Cannabis by State Legality **26**

A Substance attributes and harm data

Name	Source	Medical use	Effect	Physical harm	Psychological harm	Overall harm
Alcohol	natural	no	depressant	4th	8th	4th
Tobacco	semi-synthetic	no	depressant	3rd	25th	19th
Cannabis	natural	under research	all	21st	13th	16th
Codeine	natural	yes	depressant	23rd	22nd	22th
Heroin	semi-synthetic	no	depressant	5th	4th	3th
Cocaine	semi-synthetic	yes	stimulant	7th	3rd	5th
Xanax	synthetic	yes	depressant	16th	9th	15th
Anti-inflammatory	synthetic	yes	depressant	17th	29th	29th
Amphetamines	synthetic	yes	stimulant	11th	6th	7th
LSD	synthetic	under reseacrh	psychedelic	20th	11th	18th
Psylocibin	natural	under research	psychedelic	18th	17th	17th
MDMA	synthetic	under research	psychedelic/stimulant	15th	16th	11th
Methamphetamine	synthetic	no	stimulant	1st	1st	2nd

Supplementary Table 1: Benchmark data for real-world drug attributes manipulated in the experimental drug profiles. Data for source, medical use and effect was compiled based on publicly available information from several organizations such as the U.S. Federal Food and Drug Agency and the Drug Enforcement Agency, or the Multidisciplinary Association for Psychedelic Studies. Data for the the harm sub-components and overall harm ranks was based on an expert study (Bonnet et al, 2020).

B Pilot study

We ran a pilot survey (N=1,767) to select substances for the main studies which a large majority of US citizens are plausibly familiar with. The survey was fielded to an online opt-in panel matched to population census quotas (age, gender, race, ethnicity, region) recruited through Lucid Theorem on December 21, 2021. Informed consent was obtained. The survey was exempt from an ethical review. In the survey, we considered 24 drugs altogether, most of which were featured in the list of common psychoactive substances in the expert study we used for determining substance harms (Bonnet et al, 2020). In the survey, we asked respondents to rate these substances for legalization support (legal/illegal), or to declare if they have not heard about a substance yet. We note that the simultaneous measurement of substance familiarity and support might have biased upwards the percentage of the respondents who might be familiar with some of these drugs.

Supplementary Table 2: Mean support for legalization and proportion of "don't know substance" responses

Drug	Mean Legal	Don't know substance
Alcohol	83.5%	6.2%
Tobacco	75.2%	7.1%
Cannabis	67.9%	7.2%
Anti-Inflammatory	91.5%	8.3%
Heroin	9.5%	8.4%
Cocaine	13.3%	9.6%
Codeine	77.1%	11.4%
LSD	17.7%	16.3%
MDMA	17.7%	16.4%
Oxycontin	49.8%	17.1%
Methamphetamine	21.4%	18.6%
Amphetamines	58.8%	20.5%
Ritalin	74.6%	20.6%
Xanax	64.2%	20.9%
Psilocybin	28.5%	20.9%
Fentanyl	22.5%	22.2%
Methadone	47.5%	26.8%
Barbiturates	35.6%	30.1%
Triptane	77.9%	34.6%
Z-drugs	72.4%	37.4%
Tramadol	64.5%	40.8%
Ketamine	34.4%	46.4%
Propofol	42.4%	51.6%
GHB	27.2%	54.8%

We determined the cut-off at 75% familiarity. We decided to classify Ritalin as an Amphetamine (due to similar neurological effects), and exclude Oxycontin and Fentanyl (due to no available harm scores). This resulted in the selection of the 13 substances featured in the main study.

C Sample characteristics

Variable	Category	%	N	
Main survey				
Gender	Female	51.5	1039	
Gender	Male	48.5	977	
Age	N	–	2016	
Age	Mean (SD)	–	–	45.1 (16.7)
Age	Min–Max	–	–	18–96
Education	Less than college	51.9	1047	
Education	College degree	48.1	969	
Race	White	68.1	1372	
Race	Black	11.1	223	
Race	Latino	12.4	250	
Race	Other	8.5	171	
Party	Democrat	50.6	1020	
Party	Republican	49.4	996	
Follow-up survey				
Gender	Female	51.6	1568	
Gender	Male	48.4	1469	
Age	N	–	3037	
Age	Mean (SD)	–	–	48.3 (17.0)
Age	Min–Max	–	–	18–93
Education	Less than college degree	54.3	1650	
Education	College degree	45.7	1387	
Race	White	68.8	2089	
Race	Black	10.7	326	
Race	Latino	10.9	331	
Race	Other	9.6	291	
Party	Democrat	51.4	1561	
Party	Republican	48.6	1476	

Supplementary Table 3: Sample characteristics for the main and follow-up survey. All variables were provided by the panelist (recoded), except for partisanship which we measured during the survey (following the American National Election Study methodology: branching and forced choice). Categories that do not add up to 100% are due to missing data.

D Survey wording

D.1 Informed consent form and attention check

Procedure: You are being asked to take part in a study that examines public opinion on certain policy issues. If you decide to take part, you will complete a max. 15-minute long questionnaire. You may take the survey wherever is convenient for you.

Compensation: You will receive compensation as previously agreed with your supplier panel.

Anonymity and confidentiality: The researchers will not have personal information about you, and thus your responses will be completely anonymous. Your responses will be used in this research project only.

Outcome: Your responses will be used for statistical analyses in a research article planned for publication in social sciences. Your data will be aggregated with other responses and will not be analyzed or interpreted individually.

Voluntary participation and withdrawal: Participating in this study is entirely voluntary. If you decide to take part but change your mind later, you have the right to drop out at any time during the survey. However, keep in mind before proceeding, that all recorded responses to questions will be stored, and your consent for responding them cannot be withdrawn later.

Contact information: In case of any concerns, questions, or requests that may arise during the survey, you can contact Professor Gabor Simonovits at simonovitsg@ceu.edu.

If you are willing to participate in this research, please click on the 'AGREE' button and start the survey.

To show you are paying attention, please select "Red" and "Blue" from the items below.

- *Pink*

- *Blue*
- *Red*
- *Yellow*

D.2 Pre-treatment items

Q1. Now, we ask your opinion on some drugs you might be familiar with. Do you know anyone personally who has used the substances in the past, or you do not know anyone, or you have not heard about the substance?

– Matrix with list of selected substances –

- *I know someone*
- *I do not know anyone, but I know the substance*
- *I do not know the substance*

Q2. Now, we ask your opinion on the regulation of some common substances. Please disregard the existing legal status for a moment, and decide whether the substances below should be legal or illegal in your opinion. By legal, we mean the recreational use. You may or may not be familiar with some of the drugs displayed below. Nevertheless, we ask you to decide between the response options.

– Matrix with list of selected substances, displayed if Q1 is not 'I do not know this substance' –

- *Legal*
- *Illegal*

Q3. You answered that the substance(s) below should be illegal for recreational use. In your opinion, what would be the appropriate criminal penalty in the case of an unlawful possession of a small amount of the substance(s)? You can select between decriminalized status (illegal, but not punished by any prison sentence), misdemeanor (few months in prison), and felony (at least a year in prison).

– Matrix with list of selected substances, displayed if Q2 is 'Illegal' –

- *Decriminalized*
- *Misdemeanor*
- *Felony*

D.3 Experimental stimuli

Task introduction. You are going to read about several hypothetical substances. Please put yourself in the shoes of an expert to answer the questions related to these substances. Consider carefully all the information revealed to you in the descriptions. The following information will be made available for you about these substances:

- Source: What kind of material the substance is made of, or how it is produced.
- Therapeutic benefit: Whether the substance has the potential to be used in medical or therapeutic treatments.
- Effect: The type of reaction the substance has on the body and the brain.
- Psychological harm potential to user: The severity of the potential impairment of certain mental functioning (such as short- or long-term memory loss) resulting from consumption, and the addictiveness level of the substance. You will see the substance's psychological harm potential compared to the most harmful existing drug, on a 0-100

scale. 0 means that the described substance is not harmful at all, whereas 100 means that the described substance is as harmful as the most harmful existing drug.

- Physical harm potential to user: The severity of the possible organ and other physical damage resulting from the consumption of the substance, or from the activities related to its consumption (ingestion). You will see the substance's physical harm potential compared to the most harmful existing drug, on a 0-100 scale. 0 means that the described substance is not harmful at all, whereas 100 means that the described substance is as harmful as the most harmful existing drug.

Hypothetical drug profile screens.

– Randomized attribute levels across profiles (order of attributes fixed across profiles) –

- Source: It is manufactured from tropical plants / It has natural source but processed chemically / It is synthesized through chemical processes
- Effect: It makes you less sleepy, increases your alertness and focus, and enhances your productivity / It alters the way you see, hear, and sense things around you, it enhances your creativity, and amplifies your emotions / It makes you feel relaxed, it reduces anxiety, and the feeling of discomfort, and relieves stress and pain
- Potential psychological harm to user: a value displayed between 0-100
- Potential physical harm to user: a value displayed between 0-100

Please carefully review the description below, then please answer the question.

	Substance 1
**Production source**	It is synthesized through chemical processes.
**Therapeutic benefit**	It has no known therapeutic effects.
**Psychological harm potential to user (0-100)**	34
**Physical harm potential to user (0-100)**	0
**Effect on nervous system**	It alters the way you see, hear and sense things around you, it enhances your creativity, and amplifies your emotions.

Please decide whether the above-mentioned substance should be legal or illegal for recreational use in your opinion.

Legal | Illegal

Supplementary Figure 1: Example screen for choice task

D.4 Post-treatment items

Q5-Q9. Please decide whether the above-mentioned substance should be legal or illegal for recreational use in your opinion.

- *Legal*
- *Illegal*

Q10-Q14. What do you think should be the appropriate criminal penalty for the possession of this substance?

– Displayed immediately after questions in block Q5-Q9, if any is 'Illegal' –

- *It should be decriminalized*
- *It should be a misdemeanor*
- *It should be a felony*

E Follow-up survey wording

E.1 Pre-treatment items

Q1. Now, we ask your opinion on some drugs you might be familiar with. Do you know anyone personally who has used the substances in the past, or you do not know anyone, or you have not heard about the substance? – *Matrix with list of selected substances* –

- *I know someone*
- *I do not know anyone, but I know the substance*
- *I do not know the substance*

Q2. Now, we ask your opinion on the regulation of some common substances. Please disregard the existing legal status for a moment, and decide whether the substances below should be legal or illegal in your opinion. By legal, we mean the recreational use. – *Matrix with list of selected substances, displayed if substance is not randomized for information treatment* –

- *Legal*
- *Illegal*

Q3. You answered that the substance(s) below should be illegal for recreational use. In your opinion, what would be the appropriate criminal penalty in the case of an unlawful possession of a small amount of the substance(s)? You can select between decriminalized status (illegal, but not punished by any prison sentence), misdemeanor (few months in prison), and felony (at least a year in prison). – *Matrix with list of selected substances, displayed if Q2 is 'Illegal'* –

- *Decriminalized*

- *Misdemeanor*
- *Felony*

E.2 Subjective harm rating task

Task introduction. In the following, we are going to ask you to put yourself in the shoes of a public health expert, and evaluate a substance based on how harmful it is to those who use it. Please try to answer the questions according to the best of your knowledge even if you are unsure. We will ask you to evaluate the substance on its physical and psychological harm potential, separately:

- Physical harm to the user: The severity of the possible organ damage resulting from the drug's consumption.
- Psychological harm to the user: The severity of the possible mental impairment resulting from the drug's consumption, and the addictiveness of the drug.

You will use a 0-100 scale for the rating, where 0 represents the least harmful drug you can think of, 100 represents the most harmful drug, and 50 represents a drug with the average harmfulness compared to other substances.

Q4. In the following, we are going to ask you to rate – *Randomly selected substance from the list of those which the respondent reported familiarity with but was not selected for information treatment* –. Please use the sliders to rate this substance on the physical and psychological harm scale. Choose the value between 0-100 that you think best represents the drug's harm potential on each scale.

E.3 Experimental stimuli

Information treatment introduction. Some real public health experts also ranked substances in terms of their physical and psychological harms. We will show you the results for

one specific substance using the same 0-100 scale you used previously. Now, we will show you the expert ratings for the harm potential of – *Randomly selected substance for information treatment* – and then ask you to answer some questions.

Example of information stimuli.

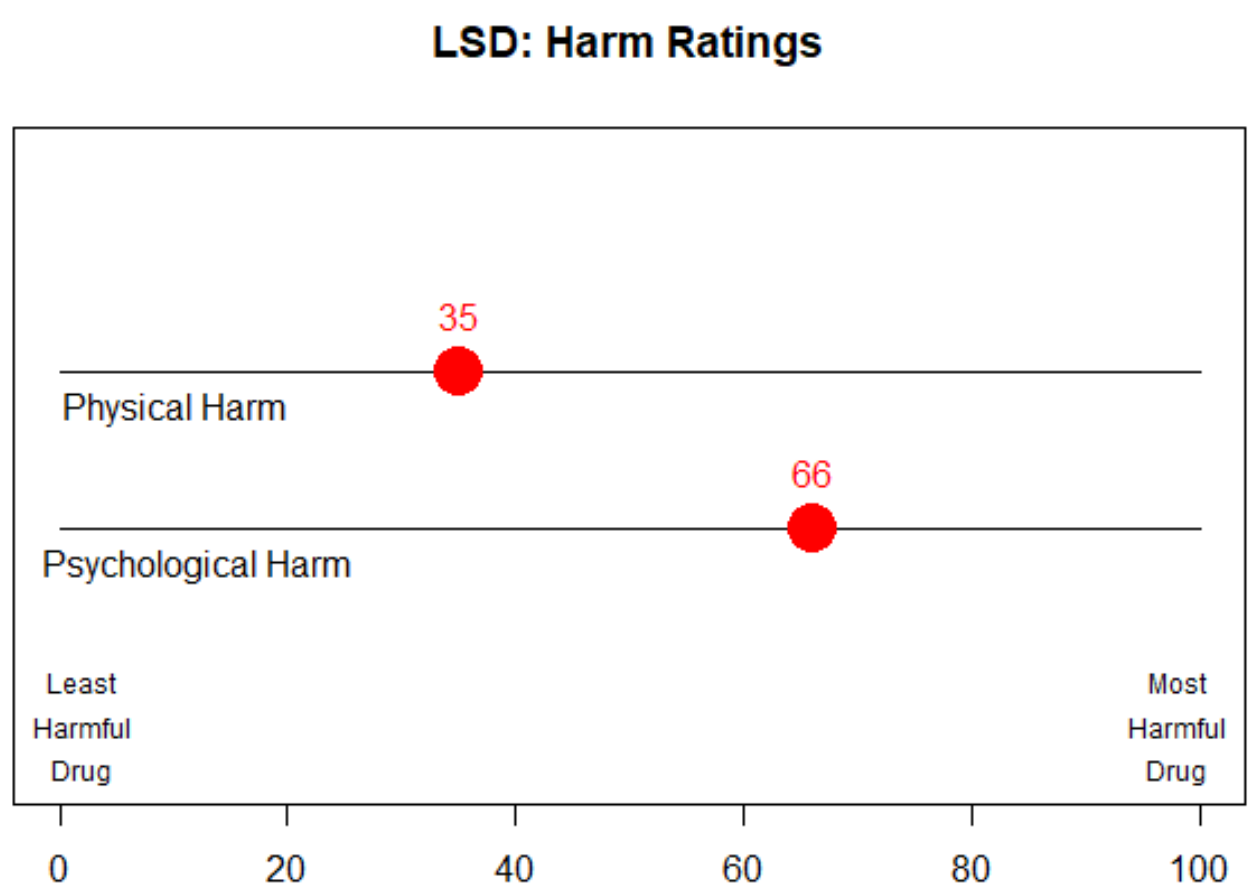

Supplementary Figure 2: Example of information stimuli screen

E.4 Post-treatment items

Q5. Please decide whether – *Displayed substance* – should be legal or illegal for recreational use in your opinion.

- *Legal*
- *Illegal*

Q6. What do you think would be the appropriate criminal penalty for the unlawful possession of – *Displayed substance* –? – *Displayed if Q5 is 'Illegal'* –

- *It should be decriminalized*
- *It should be a misdemeanor*

- *It should be a felony*

E.5 Debrief

Thank you for completing our survey!

During this survey, you have participated in an experiment, the results of which will be used in research articles on public opinion.

[Main study]: We asked you to formulate opinions on fictional substances. None of these substances were real and your opinion will NOT be taken into consideration in substance regulation. We were only curious what attributes of the drugs the public consider as important when they are deciding on legalization.

[Follow-up study]: The study explored how perceptions about substances' effects influence support for drug legalization. We asked you to rate a substance on some attributes, and also showed you a substance rated by public health experts. The latter served as an informational treatment. We were curious to know how information about drug effects influence people's regulation preferences. The information you read was actually based on public health experts' opinion, however, NOT based on established medical facts. Furthermore, there are other potentially harmful consequences, such as environmental, social or economic effects, of drug use that we did not mention here. Lastly, the potentially harmful consequences of substance use depend on the individual cases, and therefore, the information you read here should NOT serve as a consumption advice.

If you have any question, or experienced distress, please feel free to write to the principal investigator of the study at simonovitsg@ceu.edu.

F Pre-registration

The experiments' pre-analysis plans can be found in an Open Science Framework project repository using the following links:

Study 1: https://osf.io/7jn3t

Study 2: https://osf.io/h6gwb

G Attribute effects and off-brand substance support prediction model

The effect of experimental substance attributes on hypothetical legalization support, and the experimentally predicted support for the legality of off-brand drugs (certain combination of attribute levels based on expert benchmarks) was estimated relying on the following logistic regression model:

$$\begin{aligned} \text{Support(legality)} = \beta_0 + \beta_1 \cdot \text{Source} + \beta_2 \cdot \text{Medical use} + \beta_3 \cdot \text{Effect} \\ + \beta_4 \cdot \text{Psycho harm} + \beta_5 \cdot \text{Physical harm} + \epsilon_i \sim \text{Respondent} \end{aligned} \quad (1)$$

H Status quo bias tests

Supplementary Table 4: Legalization support gap between real and off-brand (experimental) illegal substances. The table shows bootstrapped mean ratings and the corresponding rating gaps (1,000 resamples, original N = 2,016), with 95% CIs in brackets, calculated from the bootstrap distribution.

	Substance	Real rating	Off-brand rating	Rating gap
1	Alcohol	86.85 [85.19, 88.40]	24.44 [21.80, 27.32]	62.41 [59.15, 65.40]
2	Amphetamines	55.51 [53.19, 57.92]	26.74 [24.06, 29.56]	28.76 [25.27, 32.53]
3	Anti-inflammatory	90.86 [89.46, 92.14]	67.11 [64.11, 70.21]	23.75 [20.46, 27.00]
4	Cannabis	73.53 [71.56, 75.53]	50.54 [39.54, 60.24]	22.99 [12.89, 34.23]
5	Cocaine	12.03 [10.58, 13.61]	24.62 [21.95, 27.42]	-12.59 [-15.44, -9.87]
6	Codeine	64.76 [62.45, 66.96]	72.93 [70.33, 75.51]	-8.16 [-11.29, -4.71]
7	Heroin	8.25 [6.99, 9.47]	21.72 [19.13, 24.51]	-13.47 [-16.46, -10.72]
8	LSD	18.20 [16.35, 19.95]	31.51 [28.61, 34.16]	-13.31 [-16.15, -10.21]
9	MDMA	16.36 [14.71, 18.18]	35.73 [28.84, 42.83]	-19.37 [-26.46, -12.30]
10	Methamphetamine	13.42 [11.73, 15.04]	10.32 [8.78, 12.06]	3.10 [0.92, 5.23]
11	Psilocybin	34.89 [32.83, 37.09]	42.66 [39.70, 45.71]	-7.77 [-11.19, -4.48]
12	Tobacco	80.59 [78.77, 82.34]	41.26 [37.58, 45.11]	39.33 [35.34, 43.20]
13	Xanax	54.92 [52.65, 57.35]	42.38 [39.40, 45.53]	12.54 [8.84, 16.08]

Supplementary Table 5: Felony support gap between real and off-brand (experimental) illegal substances. The table shows bootstrapped mean ratings and the corresponding rating gaps (1,000 resamples, original N = 2,016), with 95% CIs in brackets, calculated from the bootstrap distribution.

	Substance	Real rating	Off-brand rating	Rating gap
1	Cannabis	6.97 [5.90, 8.18]	17.63 [12.91, 23.11]	-10.66 [-16.20, -5.88]
2	Cocaine	55.85 [53.65, 58.12]	36.89 [33.19, 40.46]	18.96 [14.97, 22.71]
3	Heroin	64.48 [62.34, 66.59]	38.96 [34.90, 42.75]	25.52 [21.42, 29.66]
4	LSD	41.42 [39.13, 43.84]	27.44 [24.69, 30.41]	13.97 [10.60, 17.21]
5	MDMA	40.48 [38.17, 42.81]	25.79 [21.23, 30.65]	14.69 [9.67, 19.73]
6	Methamphetamine	52.21 [49.88, 54.56]	56.99 [52.58, 61.21]	-4.79 [-9.26, -0.33]
7	Psilocybin	26.54 [24.47, 28.69]	21.05 [18.64, 23.68]	5.49 [2.42, 8.21]

I Experimental substance attribute effects

Supplementary Table 6: Attribute effects on respondent support for experimental drug legalization (n=2,016). Estimates are from a logistic regression model (Supplementary Information G) and show the average marginal effects of independently randomized drug attribute levels in legalization support (in percentage point changes) relative to the reference level.

Term	AME	Std. Error	z-statistic	p-value	95% CI	Cohen's d
Ref: Source:Synthetic						
Source: Natural	0.061	0.0117	5.25	$< .001$	[0.038, 0.084]	0.124
Source: Semi-synthetic	0.057	0.0117	4.90	$< .001$	[0.034, 0.080]	0.116
Ref:Medical:No Medical Use						
Medical: Existing use	0.078	0.0119	6.56	$< .001$	[0.055, 0.102]	0.158
Medical: Ongoing research	0.054	0.0116	4.67	$< .001$	[0.032, 0.077]	0.110
Ref:Effect:Psychedelic						
Effect: Depressant	0.145	0.0122	11.89	$< .001$	[0.121, 0.168]	0.292
Effect: Stimulant	0.088	0.0117	7.56	$< .001$	[0.065, 0.111]	0.178
Harm:(0-100)						
Physical harm	-0.0036	0.00016	-22.41	$< .001$	[-0.0039, -0.0033]	-0.007
Psychological harm	-0.0031	0.00016	-19.28	$< .001$	[-0.0035, -0.0028]	-0.006
Representative contrasts						
Ref:High physical.harm (Alcohol)						
Low.physical.harm (Codeine)	0.236	0.011	22.41	$< .001$	[0.216, 0.257]	0.477
Mod.physical.harm (MDMA)	0.136	0.006	22.41	$< .001$	[0.124, 0.148]	0.275
Ref:High psycho harm (Cocaine)						
Low.psycho.harm (Aspirin)	0.279	0.014	19.28	$< .001$	[0.251, 0.307]	0.562
Mod.psycho.harm (Psylocibin)	0.150	0.008	19.28	$< .001$	[0.135, 0.166]	0.304
Pseudo $R^2 = 0.089$ LogLik = -6283.27 AIC = 12584.53 $N = 9990$ $df = 9981$						

J Respondent heterogeneity in experimental substabce attribute effects and the status quo bias

Descriptively, female respondents (z(9975)=-6.8, 95% CI [-9.4, -4.0], p < .001, *Cohen's d*= -0.14), White compared to Black respondents (z(9975)=6.2, 95% CI [1.9, 10.6], p < .05, *Cohen's d*= 0.13), Republicans (z(9975)=-3.8, 95% CI [-6.6, -1.1], p < .01, *Cohen's d*= -0.08), and respondents with a college degree (z(9975)=-4.8, 95% CI [-7.5, -2.1], p < .001, *Cohen's d*= -0.10) were less supportive overall in the case of hypothetical substances.

Female respondents also showed higher status quo bias, especially for prescription drugs (positive). Black respondents were more negatively biased towards illicit drugs than White

Supplementary Table 7: Attribute effects on respondent support for experimental drug legalization (n=2,016). Estimates are from a logistic regression model (Supplementary Information G) and show the average marginal effects of independently randomized drug attribute levels in legalization support (in percentage point changes) relative to the reference level.

Term	AME	Std. Error	z-statistic	p-value	95% CI	Cohen's d
Ref: Source: Synthetic						
Source: Natural	0.064	0.0117	5.53	$< .001$	[0.042, 0.087]	0.130
Source: Semi-synthetic	0.060	0.0117	5.10	$< .001$	[0.037, 0.082]	0.120
Ref: Medical: No Medical Use						
Medical: Existing use	0.078	0.0119	6.57	$< .001$	[0.055, 0.102]	0.158
Medical: Ongoing research	0.053	0.0116	4.57	$< .001$	[0.030, 0.076]	0.107
Ref: Effect: Psychedelic						
Effect: Depressant	0.146	0.0120	12.15	$< .001$	[0.123, 0.170]	0.295
Effect: Stimulant	0.089	0.0116	7.65	$< .001$	[0.066, 0.112]	0.180
Harm (0-100)						
Physical harm	-0.0035	0.00016	-22.19	$< .001$	[-0.0039, -0.0032]	-0.007
Psychological harm	-0.0032	0.00016	-19.48	$< .001$	[-0.0035, -0.0028]	-0.006
Covariates						
Female (ref: Male)	-0.067	0.0137	-4.90	$< .001$	[-0.094, -0.040]	-0.135
Black (ref: White)	0.062	0.0222	2.80	0.005	[0.019, 0.106]	0.126
Latino/a/x (ref: White)	0.020	0.0216	0.94	0.348	[-0.022, 0.063]	0.041
Other race (ref: White)	-0.023	0.0257	-0.89	0.374	[-0.073, 0.028]	-0.046
Republican (ref: Democrat)	-0.038	0.0141	-2.72	0.007	[-0.066, -0.011]	-0.077
College degree (ref: no college)	-0.048	0.0138	-3.46	$< .001$	[-0.075, -0.021]	-0.097
Pseudo $R^2 = 0.098$ LogLik = -6222.38 AIC = 12474.76 $N = 9990$ $df = 9975$						

respondents, who were more positively biased towards legal substances. Respondents with a college degree had larger positive bias for legal substances than respondents without one, but the opposite was true for illicit substances (negative). Finally, Republicans showed notably higher positive bias for legal drugs compared to Democrats.

Supplementary Table 8: Legalization support ratings by gender. The table shows bootstrapped mean ratings and the corresponding rating gaps (real–off-brand), with 95% CIs in brackets, calculated from the bootstrap distribution (1,000 resamples on N=2,016).

	Substance	Gender	Real rating	Off-brand rating	Rating gap
1	Alcohol	female	86.07 [83.94, 88.14]	22.35 [18.85, 26.02]	63.72 [59.48, 67.89]
2	Alcohol	male	87.69 [85.68, 89.86]	26.63 [22.41, 31.02]	61.07 [56.35, 65.77]
3	Amphetamines	female	57.39 [54.36, 60.71]	22.90 [19.44, 26.81]	34.49 [29.54, 39.03]
4	Amphetamines	male	53.46 [50.18, 56.81]	30.80 [26.71, 35.40]	22.66 [17.54, 27.80]
5	Anti-inflammatory	female	92.78 [91.09, 94.29]	66.09 [61.83, 70.43]	26.69 [22.26, 31.29]
6	Anti-inflammatory	male	88.75 [86.61, 90.66]	68.55 [63.99, 72.62]	20.21 [15.56, 24.84]
7	Cannabis	female	72.63 [69.97, 75.51]	47.72 [33.55, 60.36]	24.91 [12.03, 39.46]
8	Cannabis	male	74.49 [71.83, 77.25]	53.36 [43.76, 62.07]	21.13 [12.18, 31.14]
9	Cocaine	female	6.67 [5.14, 8.37]	21.16 [17.61, 25.20]	-14.49 [-18.28, -10.91]
10	Cocaine	male	17.68 [15.27, 20.28]	28.37 [24.42, 32.69]	-10.69 [-15.04, -6.44]
11	Codeine	female	64.98 [61.99, 67.80]	72.99 [69.27, 76.63]	-8.01 [-12.42, -3.35]
12	Codeine	male	64.52 [61.24, 67.65]	73.28 [69.55, 76.80]	-8.76 [-12.98, -4.31]
13	Heroin	female	5.21 [3.87, 6.58]	19.34 [15.91, 23.01]	-14.13 [-17.92, -10.70]
14	Heroin	male	11.54 [9.51, 13.68]	24.16 [19.96, 28.75]	-12.62 [-17.14, -8.00]
15	LSD	female	11.40 [9.08, 13.46]	25.78 [22.42, 29.19]	-14.38 [-17.86, -10.64]
16	LSD	male	24.98 [22.21, 27.97]	37.30 [33.03, 41.61]	-12.33 [-16.63, -7.47]
17	MDMA	female	10.67 [8.64, 12.80]	31.03 [22.67, 39.89]	-20.36 [-29.45, -11.68]
18	MDMA	male	22.33 [19.66, 25.40]	40.55 [33.59, 47.74]	-18.22 [-25.78, -10.90]
19	Methamphetamine	female	9.75 [7.95, 11.88]	8.13 [6.22, 10.12]	1.62 [-0.87, 4.30]
20	Methamphetamine	male	17.27 [14.42, 19.90]	12.81 [10.04, 16.01]	4.46 [1.03, 7.70]
21	Psylocibin	female	28.33 [25.33, 31.14]	37.55 [33.46, 41.94]	-9.22 [-14.15, -4.58]
22	Psylocibin	male	41.53 [38.25, 44.99]	47.75 [43.46, 51.90]	-6.21 [-10.93, -1.54]
23	Tobacco	female	78.44 [75.77, 80.94]	39.35 [34.47, 44.29]	39.09 [33.83, 44.61]
24	Tobacco	male	82.92 [80.57, 85.22]	43.59 [38.66, 48.73]	39.32 [33.54, 44.75]
25	Xanax	female	57.02 [53.67, 60.22]	39.54 [35.02, 43.87]	17.48 [12.18, 22.85]
26	Xanax	male	52.56 [49.29, 56.02]	45.19 [40.71, 49.57]	7.37 [1.98, 12.88]

Supplementary Table 9: Legalization support ratings by race. The table shows bootstrapped mean ratings and the corresponding rating gaps (real–off-brand), with 95% CIs in brackets, calculated from the bootstrap distribution (1,000 resamples on N=2,016).

	Substance	Race	Real rating	Off-brand rating	Rating gap
1	Alcohol	Black	89.05 [84.75, 92.97]	40.67 [30.02, 51.62]	48.39 [37.00, 59.74]
2	Alcohol	Latino/a/x	80.53 [75.52, 85.91]	27.60 [20.05, 36.35]	52.93 [43.23, 62.03]
3	Alcohol	Other	83.72 [78.02, 88.97]	15.15 [9.04, 23.32]	68.56 [58.75, 76.59]
4	Alcohol	White	88.01 [86.13, 89.86]	23.02 [19.86, 26.61]	64.98 [60.81, 68.70]
5	Amphetamines	Black	55.80 [49.02, 62.72]	31.95 [22.95, 42.08]	23.85 [12.54, 35.40]
6	Amphetamines	Latino/a/x	55.13 [48.51, 61.54]	29.35 [21.46, 37.79]	25.78 [15.61, 35.43]
7	Amphetamines	Other	46.26 [38.24, 54.75]	22.46 [14.75, 31.68]	23.79 [12.23, 34.17]
8	Amphetamines	White	56.62 [53.92, 59.40]	25.65 [22.40, 28.95]	30.97 [27.04, 35.31]
9	Anti-inflammatory	Black	85.23 [79.51, 89.89]	70.90 [61.46, 79.04]	14.33 [4.37, 24.71]
10	Anti-inflammatory	Latino/a/x	84.55 [79.91, 88.79]	64.01 [53.84, 72.86]	20.54 [10.35, 31.16]
11	Anti-inflammatory	Other	87.79 [82.12, 92.41]	62.90 [50.73, 73.39]	24.88 [13.90, 37.35]
12	Anti-inflammatory	White	93.23 [91.79, 94.54]	67.82 [64.22, 71.39]	25.41 [21.82, 29.53]
13	Cannabis	Black	77.99 [72.48, 83.58]	59.07 [43.74, 73.38]	18.92 [3.29, 35.15]
14	Cannabis	Latino/a/x	79.15 [73.66, 84.28]	50.64 [38.98, 63.74]	28.51 [14.88, 40.88]
15	Cannabis	Other	68.62 [61.17, 75.46]	40.94 [27.00, 53.98]	27.68 [12.94, 42.90]
16	Cannabis	White	72.42 [70.02, 74.82]	50.42 [37.84, 61.38]	22.00 [10.77, 34.81]
17	Cocaine	Black	11.39 [6.99, 16.13]	32.32 [23.17, 42.35]	-20.92 [-30.49, -11.81]
18	Cocaine	Latino/a/x	10.84 [6.91, 14.98]	28.83 [20.75, 37.77]	-17.99 [-27.20, -9.28]
19	Cocaine	Other	10.73 [6.29, 15.89]	23.79 [14.19, 34.60]	-13.05 [-24.49, -3.15]
20	Cocaine	White	12.49 [10.79, 14.36]	22.58 [19.60, 25.95]	-10.08 [-13.37, -6.78]
21	Codeine	Black	54.32 [46.73, 61.70]	78.29 [71.95, 84.52]	-23.97 [-33.82, -14.53]
22	Codeine	Latino/a/x	57.94 [51.12, 64.12]	68.58 [60.40, 75.92]	-10.64 [-20.34, -0.16]
23	Codeine	Other	60.99 [52.76, 68.36]	68.85 [57.68, 78.01]	-7.87 [-20.85, 4.83]
24	Codeine	White	67.97 [65.44, 70.36]	73.64 [70.50, 76.58]	-5.68 [-9.37, -1.84]
25	Heroin	Black	6.51 [3.11, 10.58]	33.73 [23.23, 43.84]	-27.22 [-37.93, -16.73]
26	Heroin	Latino/a/x	12.15 [8.26, 16.52]	30.12 [21.27, 39.96]	-17.97 [-28.22, -8.40]

Continued on next page

27	Heroin	Other	10.21 [5.77, 15.39]	15.09 [8.34, 23.99]	-4.89 [-13.78, 2.88]
28	Heroin	White	7.56 [6.09, 9.02]	19.57 [16.72, 22.76]	-12.01 [-15.23, -8.67]
29	LSD	Black	12.88 [7.64, 18.54]	34.87 [27.55, 42.60]	-21.99 [-31.41, -13.30]
30	LSD	Latino/a/x	21.40 [15.88, 27.66]	40.56 [32.52, 48.62]	-19.17 [-28.61, -9.70]
31	LSD	Other	19.27 [12.39, 26.53]	23.61 [15.27, 33.61]	-4.34 [-13.81, 4.11]
32	LSD	White	18.27 [16.21, 20.42]	30.15 [26.81, 33.60]	-11.88 [-15.43, -8.45]
33	MDMA	Black	18.07 [12.81, 23.93]	40.95 [29.54, 53.77]	-22.88 [-36.98, -10.70]
34	MDMA	Latino/a/x	19.55 [14.36, 24.88]	39.14 [31.32, 47.45]	-19.59 [-29.24, -10.21]
35	MDMA	Other	14.06 [8.47, 20.16]	28.56 [17.01, 41.59]	-14.50 [-28.44, -2.25]
36	MDMA	White	15.81 [13.79, 17.93]	35.02 [26.96, 43.17]	-19.21 [-27.61, -11.20]
37	Methamphetamine	Black	17.26 [11.33, 23.21]	16.58 [10.26, 23.96]	0.68 [-8.01, 8.58]
38	Methamphetamine	Latino/a/x	18.34 [12.92, 23.62]	13.91 [8.49, 20.51]	4.44 [-4.27, 12.03]
39	Methamphetamine	Other	15.16 [9.68, 20.97]	6.96 [3.21, 12.08]	8.20 [1.37, 14.69]
40	Methamphetamine	White	11.83 [10.07, 13.53]	9.24 [7.51, 11.30]	2.60 [0.27, 4.87]
41	Psylocibin	Black	32.16 [25.32, 39.62]	51.62 [43.17, 60.09]	-19.47 [-30.63, -9.20]
42	Psylocibin	Latino/a/x	36.21 [29.95, 42.49]	45.48 [37.16, 54.69]	-9.27 [-18.63, 0.13]
43	Psylocibin	Other	33.07 [24.81, 41.13]	36.13 [25.89, 47.30]	-3.06 [-15.51, 7.37]
44	Psylocibin	White	35.25 [32.59, 37.82]	41.55 [38.04, 45.44]	-6.30 [-10.71, -2.31]
45	Tobacco	Black	81.93 [76.39, 87.19]	55.86 [44.40, 66.38]	26.06 [15.01, 38.35]
46	Tobacco	Latino/a/x	75.77 [70.26, 81.62]	43.28 [33.49, 53.00]	32.48 [21.72, 44.18]
47	Tobacco	Other	68.44 [61.33, 75.41]	37.37 [25.64, 50.11]	31.07 [18.00, 44.91]
48	Tobacco	White	82.72 [80.70, 84.69]	39.31 [34.90, 43.92]	43.41 [38.49, 48.07]
49	Xanax	Black	52.44 [45.55, 59.43]	48.25 [37.94, 58.14]	4.19 [-8.28, 16.20]
50	Xanax	Latino/a/x	48.54 [42.02, 55.00]	47.32 [38.48, 56.32]	1.22 [-9.01, 11.40]
51	Xanax	Other	43.41 [35.21, 51.41]	31.48 [22.32, 41.51]	11.93 [-0.79, 24.81]
52	Xanax	White	57.73 [55.02, 60.59]	41.84 [38.12, 45.44]	15.89 [11.54, 20.40]

Supplementary Table 10: Legalization support ratings by education level. The table shows bootstrapped mean ratings and the corresponding rating gaps (real–off-brand), with 95% CIs in brackets, calculated from the bootstrap distribution (1,000 resamples on N=2,016)

	Substance	Education	Real rating	Off-brand rating	Rating gap
1	Alcohol	college degree	89.17 [86.98, 91.21]	19.15 [15.78, 23.01]	70.02 [65.63, 74.03]
2	Alcohol	less than college degree	84.67 [82.41, 86.94]	29.64 [25.47, 33.83]	55.03 [50.07, 59.63]
3	Amphetamines	college degree	57.17 [53.95, 60.29]	24.01 [20.38, 27.94]	33.16 [28.28, 38.37]
4	Amphetamines	less than college degree	53.93 [50.87, 57.24]	29.23 [25.09, 33.29]	24.69 [19.87, 29.38]
5	Anti-inflammatory	college degree	91.63 [89.67, 93.38]	67.05 [62.42, 71.18]	24.58 [20.09, 29.44]
6	Anti-inflammatory	less than college degree	90.14 [88.23, 91.92]	67.37 [63.13, 71.56]	22.77 [18.35, 27.53]
7	Cannabis	college degree	70.21 [67.35, 73.18]	45.65 [33.11, 55.67]	24.57 [14.14, 37.24]
8	Cannabis	less than college degree	76.66 [74.04, 79.27]	54.90 [43.47, 65.87]	21.76 [10.53, 33.36]
9	Cocaine	college degree	12.11 [10.07, 14.39]	21.99 [18.34, 25.80]	-9.88 [-13.64, -6.13]
10	Cocaine	less than college degree	11.96 [9.91, 13.98]	27.07 [22.98, 31.24]	-15.11 [-19.23, -10.93]
11	Codeine	college degree	68.03 [64.83, 70.99]	70.49 [66.58, 74.18]	-2.46 [-7.17, 2.08]
12	Codeine	less than college degree	61.65 [58.44, 64.81]	75.23 [71.85, 78.45]	-13.58 [-17.75, -8.95]
13	Heroin	college degree	8.27 [6.60, 10.22]	17.79 [14.41, 21.45]	-9.52 [-13.22, -6.22]
14	Heroin	less than college degree	8.24 [6.58, 9.95]	25.35 [21.49, 29.48]	-17.12 [-21.38, -12.61]
15	LSD	college degree	18.77 [16.22, 21.40]	27.65 [23.69, 31.63]	-8.88 [-13.12, -4.79]
16	LSD	less than college degree	17.63 [15.09, 20.33]	34.83 [30.80, 38.76]	-17.20 [-21.14, -13.16]
17	MDMA	college degree	15.89 [13.41, 18.46]	33.48 [24.65, 42.45]	-17.59 [-26.72, -8.88]
18	MDMA	less than college degree	16.82 [14.25, 19.43]	37.67 [30.50, 44.99]	-20.85 [-28.49, -13.37]
19	Methamphetamine	college degree	14.39 [11.95, 16.79]	8.39 [6.47, 10.59]	6.01 [3.30, 8.87]
20	Methamphetamine	less than college degree	12.52 [10.35, 14.88]	12.16 [9.60, 15.05]	0.36 [-2.90, 3.55]
21	Psylocibin	college degree	32.90 [29.69, 36.22]	37.92 [33.73, 42.04]	-5.02 [-10.08, -0.41]
22	Psylocibin	less than college degree	36.83 [33.61, 40.05]	46.94 [42.65, 51.15]	-10.11 [-14.91, -5.46]
23	Tobacco	college degree	80.34 [77.68, 82.91]	39.99 [35.17, 45.12]	40.35 [34.76, 46.22]
24	Tobacco	less than college degree	80.83 [78.34, 83.27]	42.36 [37.42, 47.43]	38.47 [33.08, 43.66]
25	Xanax	college degree	58.42 [55.22, 61.57]	37.77 [33.22, 42.46]	20.65 [15.16, 25.85]
26	Xanax	less than college degree	51.64 [48.35, 54.95]	46.38 [41.95, 50.71]	5.25 [0.33, 10.27]

Supplementary Table 11: Legalization support ratings by partisanship (forced). The table shows bootstrapped mean ratings and the corresponding rating gaps (real–off-brand), with 95% CIs in brackets, calculated from the bootstrap distribution (1,000 resamples on N=2,016)

	Substance	Party	Real rating	Off-brand rating	Rating gap
1	Alcohol	Democrat	87.63 [85.39, 89.74]	26.42 [22.44, 30.68]	61.22 [56.63, 65.61]
2	Alcohol	Republican	86.06 [83.61, 88.40]	22.67 [18.94, 26.85]	63.40 [58.59, 67.94]
3	Amphetamines	Democrat	57.73 [54.47, 60.92]	26.75 [23.10, 30.30]	30.98 [26.43, 36.03]
4	Amphetamines	Republican	53.29 [49.83, 56.70]	26.58 [22.66, 30.89]	26.71 [21.65, 31.54]
5	Anti-inflammatory	Democrat	90.39 [88.43, 92.16]	71.16 [66.86, 74.98]	19.23 [14.99, 23.67]
6	Anti-inflammatory	Republican	91.33 [89.42, 93.07]	63.13 [58.68, 67.75]	28.20 [23.46, 33.13]
7	Cannabis	Democrat	80.37 [77.67, 82.91]	53.24 [41.33, 65.29]	27.12 [14.87, 39.10]
8	Cannabis	Republican	66.61 [63.53, 69.46]	47.72 [35.68, 56.88]	18.89 [9.27, 31.23]
9	Cocaine	Democrat	13.41 [11.16, 15.49]	24.38 [20.74, 28.09]	-10.98 [-14.81, -7.12]
10	Cocaine	Republican	10.66 [8.66, 12.61]	24.79 [20.79, 29.23]	-14.13 [-18.53, -10.01]
11	Codeine	Democrat	64.45 [61.36, 67.55]	76.07 [72.48, 79.14]	-11.62 [-16.03, -7.06]
12	Codeine	Republican	65.07 [61.89, 68.11]	69.93 [66.01, 73.86]	-4.86 [-9.54, 0.06]
13	Heroin	Democrat	9.08 [7.37, 10.88]	23.83 [19.92, 28.21]	-14.75 [-19.31, -10.58]
14	Heroin	Republican	7.42 [5.82, 9.09]	19.81 [16.40, 23.41]	-12.39 [-16.41, -8.79]
15	LSD	Democrat	20.76 [17.98, 23.51]	34.45 [30.64, 38.45]	-13.69 [-18.09, -9.51]
16	LSD	Republican	15.66 [12.99, 18.18]	28.36 [24.59, 32.15]	-12.70 [-17.07, -8.37]
17	MDMA	Democrat	17.36 [14.96, 20.00]	37.89 [30.87, 45.25]	-20.53 [-28.12, -13.23]
18	MDMA	Republican	15.37 [12.83, 17.91]	33.34 [25.03, 42.36]	-17.97 [-26.98, -9.16]
19	Methamphetamine	Democrat	13.71 [11.44, 16.02]	10.06 [7.80, 12.40]	3.65 [0.73, 6.44]
20	Methamphetamine	Republican	13.14 [10.90, 15.24]	10.45 [8.21, 13.07]	2.69 [-0.44, 5.63]
21	Psylocibin	Democrat	39.15 [36.07, 42.34]	45.60 [41.38, 49.91]	-6.45 [-11.40, -1.78]
22	Psylocibin	Republican	30.69 [27.84, 33.70]	39.57 [35.43, 44.00]	-8.88 [-13.71, -4.32]
23	Tobacco	Democrat	79.68 [77.14, 82.26]	45.04 [39.92, 50.07]	34.64 [28.88, 40.46]
24	Tobacco	Republican	81.52 [79.18, 84.06]	37.85 [33.28, 43.04]	43.67 [38.34, 48.75]
25	Xanax	Democrat	57.35 [54.26, 60.96]	46.14 [41.84, 50.79]	11.21 [6.13, 16.19]
26	Xanax	Republican	52.50 [49.23, 56.01]	38.84 [34.54, 43.27]	13.66 [8.50, 19.24]

K Harm information effects

Supplementary Table 12: Baseline misperception of drug harms. Table reports the mean subjective harm ratings with 95% confidence intervals, the expert harm scores, and their gap (subjective – expert). One-sample, two-tailed t-tests were performed to test whether the gap between the subjective and expert ratings differs from zero. No multiple comparison corrections were made as the drug-level hypotheses were tested on independent sub-samples.

Drug	Subjective Mean	95% CI	Expert Score	Mean Diff	t-statistic	p-value	n
Alcohol	65.564	[63.412, 67.715]	83.000	-17.436	-15.931	$< .001$	393
Cocaine	81.043	[79.394, 82.693]	86.500	-5.457	-6.503	$< .001$	392
LSD	68.874	[66.457, 71.291]	50.500	18.374	14.946	$< .001$	409
Cannabis	32.743	[30.219, 35.267]	45.000	-12.257	-9.547	$< .001$	407
MDMA	70.600	[68.396, 72.803]	50.000	20.600	18.374	$< .001$	412
Tobacco	60.261	[58.047, 62.475]	55.000	5.261	4.672	$< .001$	393
Xanax	56.617	[54.222, 59.012]	60.000	-3.383	-2.777	0.006	397

Supplementary Table 13: Harm information effect on drug legalization support. Table shows the results of two-sample, two-tailed t-tests comparing the mean difference in legalization support (binary outcome) between the control (no harm information) and harm information treatment group by substance. No multiple comparison corrections were made as the drug-level hypotheses were tested on independent sub-samples.

Drug	Control Mean	Informed Mean	Mean Diff	95% CI	t-statistic	p-value	Cohen's d	Control n	Informed n
Alcohol	0.859	0.682	-0.177	[-0.223, -0.131]	7.48	$< .001$	-0.476	2610	424
Cocaine	0.109	0.128	0.019	[-0.015, 0.053]	-1.11	0.266	0.061	2598	436
LSD	0.159	0.197	0.038	[-0.002, 0.078]	-1.84	0.065	0.102	2598	432
Cannabis	0.706	0.680	-0.026	[-0.073, 0.022]	1.06	0.289	-0.056	2599	435
MDMA	0.147	0.182	0.035	[-0.004, 0.074]	-1.76	0.078	0.098	2604	428
Tobacco	0.780	0.651	-0.129	[-0.177, -0.081]	5.31	$< .001$	-0.303	2596	433
Xanax	0.465	0.228	-0.237	[-0.281, -0.194]	10.70	$< .001$	-0.479	2585	447

Supplementary Table 14: Harm information effect on misperception of drug harms. Table shows the results of two-sample, two-tailed t-tests comparing the mean difference in harm misperception (subjective harm rating – expert harm rating, 0–100) between the control (no harm information) and the harm information treatment group by substance. To keep potential outcomes equal between groups, we only use observations from the treated group in cases when the respondent was familiar with the given substance pre-treatment. No multiple comparison corrections were made as the drug-level hypotheses were tested on independent sub-samples.

Drug	Control Mean	Informed Mean	Mean Diff	95% CI	t-statistic	p-value	Control n	Informed n
Alcohol	-17.436	-10.594	6.842	[4.011, 9.673]	-4.75	$< .001$	393	387
Cocaine	-5.457	-3.844	1.613	[-0.775, 4.001]	-1.33	0.186	392	368
LSD	18.374	16.134	-2.240	[-5.498, 1.018]	1.35	0.178	409	347
Cannabis	-12.257	-3.254	9.003	[5.343, 12.663]	-4.83	$< .001$	407	394
MDMA	20.600	17.355	-3.244	[-6.292, -0.197]	2.09	0.037	412	318
Tobacco	5.261	7.557	2.297	[-0.642, 5.235]	-1.53	0.126	393	401
Xanax	-3.383	2.855	6.238	[3.194, 9.282]	-4.02	$< .001$	397	342

L Status quo bias moderation by harm information

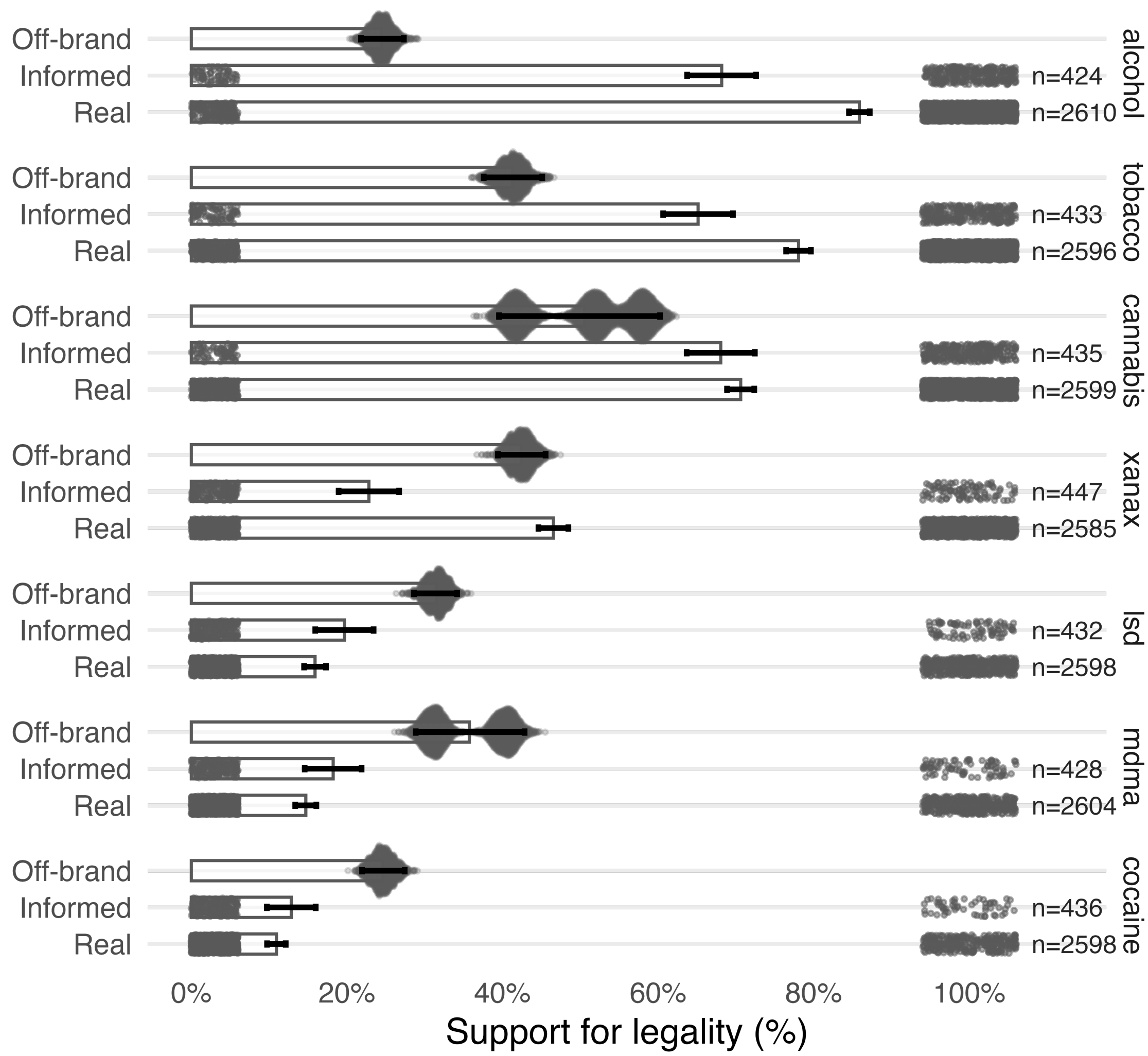

Supplementary Figure 3: Comparison of legalization support under harm information, no information, and for off-brand drugs. Real (control group) and informed mean respondent support is drawn from Study 2. Off-brand mean respondent support (experimental predictions) is based on Study 1 and estimated via bootstrapping (1,000 resamples, original n = 2,016 respondents). Bars show mean absolute support by rating type. Error bars denote 95% confidence intervals; off-brand intervals are based on the bootstrap sample distribution. Overlaid data show raw binary responses for real and informed ratings and the bootstrap mean distribution for off-brand ratings.

M Status quo bias for Cannabis by State Legality

We also pre-registered an analysis for how the size of the status quo bias varies for Cannabis, a substance which has mixed legal status across states. We subset the sample by respondents' self-reported state of residence, and calculate the real support, the off-brand support, and their difference for these sub samples. We report the findings of this analysis in Supplementary Figure 4 below.

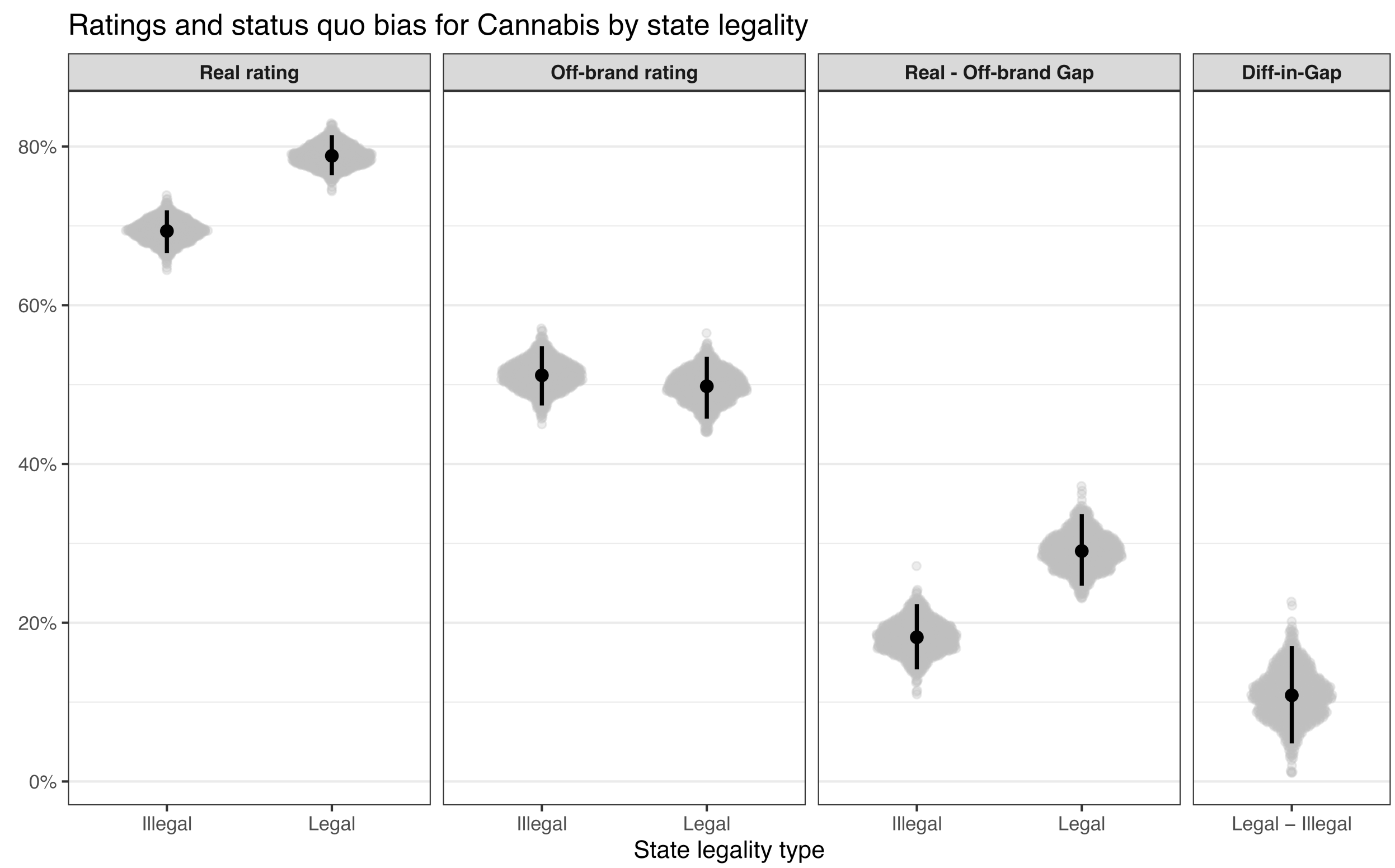

Supplementary Figure 4: Comparison of respondent legalization support for real and off-brand Cannabis in states where the substance is legal or illegal for recreational use. Points estimates show mean support per rating type, or their difference. The third column shows the mean difference between real and off-brand rating of Cannabis legalization per state type. The fourth column shows the difference-in-the-rating-differences between state types (legal minus illegal state). Real and off-brand respondent support (experimental predictions) are estimated via bootstrapping (1,000 resamples, original n = 2,016 respondents). Error bars denote 95% confidence intervals based on the bootstrap sample distribution. Overlaid data show the bootstrap mean distribution for a rating, or their difference.
