# Supplementary material for: Status quo bias drives public support for drug policy in the USA
Source: Nat Hum Behav. 2026 May 20;10(8):1487–94. doi: 10.1038/s41562-026-02471-y (PMC13485701; doi:10.1038/s41562-026-02471-y)
Supplement: Supplementary file 2 [file 41562_2026_2471_MOESM2_ESM.pdf]

nature portfolio

Corresponding author(s): Gabor Simonovits and Bence Hamrak

Last updated by author(s): 20 March 2026

Reporting Summary

Nature Portfolio wishes to improve the reproducibility of the work that we publish. This form provides structure for consistency and transparency in reporting. For further information on Nature Portfolio policies, see our Editorial Policies and the Editorial Policy Checklist.

Statistics

For all statistical analyses, confirm that the following items are present in the figure legend, table legend, main text, or Methods section.

n/a	Confirmed	
☐	☒	The exact sample size (*n*) for each experimental group/condition, given as a discrete number and unit of measurement
☐	☒	A statement on whether measurements were taken from distinct samples or whether the same sample was measured repeatedly
☐	☒	The statistical test(s) used AND whether they are one- or two-sided<br>*Only common tests should be described solely by name; describe more complex techniques in the Methods section.*
☐	☒	A description of all covariates tested
☐	☒	A description of any assumptions or corrections, such as tests of normality and adjustment for multiple comparisons
☐	☒	A full description of the statistical parameters including central tendency (e.g. means) or other basic estimates (e.g. regression coefficient) AND variation (e.g. standard deviation) or associated estimates of uncertainty (e.g. confidence intervals)
☐	☒	For null hypothesis testing, the test statistic (e.g. *F*, *t*, *r*) with confidence intervals, effect sizes, degrees of freedom and *P* value noted<br>*Give P values as exact values whenever suitable.*
☒	☐	For Bayesian analysis, information on the choice of priors and Markov chain Monte Carlo settings
☒	☐	For hierarchical and complex designs, identification of the appropriate level for tests and full reporting of outcomes
☐	☒	Estimates of effect sizes (e.g. Cohen's *d*, Pearson's *r*), indicating how they were calculated

Our web collection on statistics for biologists contains articles on many of the points above.

Software and code

Policy information about availability of computer code

Data collection

Data was collected by Lucid Theorem through a Qualtrics link provided by the researchers.

Data analysis

```
R version 4.4.2 (2024-10-31)
Platform: aarch64-apple-darwin20
Running under: macOS 26.2

Matrix products: default
LAPACK: /Library/Frameworks/R.framework/Versions/4.4-arm64/Resources/lib/libRlapack.dylib;  LAPACK version 3.12.0

locale:
[1] en_US.UTF-8/en_US.UTF-8/en_US.UTF-8/C/en_US.UTF-8/en_US.UTF-8

attached base packages:
[1] stats     graphics  grDevices utils     datasets  methods   base

other attached packages:
 [1] ggbeeswarm_0.7.2 patchwork_1.3.0  ggrepel_0.9.6    knitr_1.49       lubridate_1.9.4
 [6] forcats_1.0.0    readr_2.1.5      ggplot2_3.5.1    tidyverse_2.0.0  broom_1.0.7
[11] sandwich_3.1-1   lmtest_0.9-40    zoo_1.8-12       margins_0.3.28   arm_1.14-4
[16] lme4_1.1-35.5    Matrix_1.7-1     MASS_7.3-61      here_1.0.1       purrr_1.0.2
```

```
[21] scales_1.3.0    tibble_3.2.1    rsample_1.2.1   stringi_1.8.4   stringr_1.5.1
[26] tidyr_1.3.1     haven_2.5.4     foreign_0.8-87  dplyr_1.1.4
```

For manuscripts utilizing custom algorithms or software that are central to the research but not yet described in published literature, software must be made available to editors and reviewers. We strongly encourage code deposition in a community repository (e.g. GitHub). See the Nature Portfolio guidelines for submitting code & software for further information.

Data

Policy information about availability of data

All manuscripts must include a data availability statement. This statement should provide the following information, where applicable:

- Accession codes, unique identifiers, or web links for publicly available datasets
- A description of any restrictions on data availability
- For clinical datasets or third party data, please ensure that the statement adheres to our policy

All data and code necessary to reproduce the analyses are available https://doi.org/10.5281/zenodo.19130713

Research involving human participants, their data, or biological material

Policy information about studies with human participants or human data. See also policy information about sex, gender (identity/presentation), and sexual orientation and race, ethnicity and racism.

Reporting on sex and gender	The Supplementary Information details descriptive statistics on gender (self-reported). Furthermore, we share results for the analyses of drug attribute effects and status quo bias when gender is controlled for, and also subset results by gender. See Supplementary Information Table 3., Table 7., and Table 8.
Reporting on race, ethnicity, or other socially relevant groupings	The Supplementary Information details descriptive statistics on race (self-reported). Race categories were determined by the sample provider, Lucid Theorem. Furthermore, we share results for the analyses of drug attribute effects and status quo bias when gender is controlled for, and also subset results by gender. See Supplementary Information Table 3., Table 7. and Table 9.
Population characteristics	Our panel provided information about gender, age, education, race. See Supplementary Information Table 3.
Recruitment	Conducted by Lucid Theorem, an online opt-in panel
Ethics oversight	The studies received an ethical approval from the Ethics Committee of the Central European University (2022-2023/6/EX).

Note that full information on the approval of the study protocol must also be provided in the manuscript.

Field-specific reporting

Please select the one below that is the best fit for your research. If you are not sure, read the appropriate sections before making your selection.

☐ Life sciences ☒ Behavioural & social sciences ☐ Ecological, evolutionary & environmental sciences

For a reference copy of the document with all sections, see nature.com/documents/nr-reporting-summary-flat.pdf

Behavioural & social sciences study design

All studies must disclose on these points even when the disclosure is negative.

Study description	Main study: A quantitative conjoint experiment with hypothetical substance profiles and rating exercises.<br>Follow up: A quantitative survey experiment where each participant was randomly exposed to information or no information for a given substance and their harm perceptions and support were measured.
Research sample	Online opt-in sample quota-matched to US population census on age, gender, race, ethnicity, geographic regions.
Sampling strategy	Random sampling. The sample sizes were determined based on the sample sizes and effect sizes in previous relevant publications.
Data collection	Data was collected by Lucid Theorem through the Qualtrics link we provided. Data collection was double-blind.
Timing	main survey: July 21, 2023<br>follow-up survey: April 23, 2024
Data exclusions	We screened respondents with attention checks in the beginning of the surveys.<br>main study: 82% passed (2016 out of 2460 attempted)<br>follow up study: 83,4% passed (3037 out of 3638)<br>Inattentive respondents were eliminated from the survey before answering further questions.

Non-participation	No respondents who entered the survey declined participation.
Randomization	Respondents were randomly allocated to experimental groups.

Reporting for specific materials, systems and methods

We require information from authors about some types of materials, experimental systems and methods used in many studies. Here, indicate whether each material, system or method listed is relevant to your study. If you are not sure if a list item applies to your research, read the appropriate section before selecting a response.

Materials & experimental systems

n/a	Involved in the study
☒	☐ Antibodies
☒	☐ Eukaryotic cell lines
☒	☐ Palaeontology and archaeology
☒	☐ Animals and other organisms
☒	☐ Clinical data
☒	☐ Dual use research of concern
☒	☐ Plants

Methods

n/a	Involved in the study
☒	☐ ChIP-seq
☒	☐ Flow cytometry
☒	☐ MRI-based neuroimaging

Plants

Seed stocks	n.a.
Novel plant genotypes	n.a.
Authentication	n.a.
